# Association between the HFE C282Y, H63D Polymorphisms and the Risks of Non-Alcoholic Fatty Liver Disease, Liver Cirrhosis and Hepatocellular Carcinoma: An Updated Systematic Review and Meta-Analysis of 5,758 Cases and 14,741 Controls

**DOI:** 10.1371/journal.pone.0163423

**Published:** 2016-09-22

**Authors:** Qing Ye, Bao-Xin Qian, Wei-Li Yin, Feng-Mei Wang, Tao Han

**Affiliations:** 1 The Third Central clinical college of Tianjin Medical University, Tianjin, PR China; 2 Department of Gastroenterology and Hepatology, Tianjin Third Central Hospital, Tianjin, PR China; 3 Tianjin Institute of Hepatobiliary Disease, Tianjin, PR China; 4 Tianjin Key Laboratory of Artificial Cells, Tianjin, PR China; Medizinische Fakultat der RWTH Aachen, GERMANY

## Abstract

**Background:**

Conflicting results have been obtained for the association between two common polymorphisms (C282Y, H63D) of human HFE (hereditary hemochromatosis) gene and the risks of the liver diseases, including non-alcoholic fatty liver disease (NAFLD), liver cirrhosis and hepatocellular carcinoma (HCC).

**Methods:**

An updated systematic review and meta-analysis was conducted to evaluate the potential role of HFE polymorphisms in the susceptibility to NAFLD, liver cirrhosis and HCC. After retrieving articles from online databases, eligible studies were enrolled according to the selection criteria. Stata/SE 12.0 software was utilized to perform the statistical analysis.

**Results:**

In total, 43 articles with 5,758 cases and 14,741 controls were selected. Compared with the control group, a significantly increased risk of NAFLD was observed for the C282Y polymorphism in the Caucasian population under all genetic models and for the H63D polymorphism under the allele, heterozygote and dominant models (all OR>1, *P*_association_<0.05). However, no significant difference between liver cirrhosis cases and the control group was observed for HFE C282Y and H63D (all *P*_association_>0.05). In addition, we found that HFE C282Y was statistically associated with increased HCC susceptibility in the overall population, while H63D increased the odds of developing non-cirrhotic HCC in the African population (all OR>1, *P*_association_<0.05). Moreover, a positive association between compound heterozygosity for C282Y/H63D and the risk of NAFLD and HCC, but not liver cirrhosis, was observed.

**Conclusion:**

Our meta-analysis provides evidence that the HFE C282Y and H63D polymorphisms confer increased genetic susceptibility to NAFLD and HCC but not liver cirrhosis. Additional well-powered studies are required to confirm our conclusion.

## Introduction

Hepatocellular carcinoma (HCC) often occurs as the end-stage or more aggressive form of many progressive chronic liver diseases, such as NAFLD (non-alcoholic fatty liver disease), liver cirrhosis, and chronic viral hepatitis [1–4]. NAFLD is a series of chronic liver disease with fat deposition in the absence of significant alcohol consumption, including simple fatty liver, NASH (non-alcoholic steatohepatitis), liver fibrosis and cirrhosis [5, 6]. Liver cirrhosis is a common clinical chronic liver injury that is characterized by the formation of microscopic or macroscopic nodules separated by bands of fibrous tissue, impairment of hepatocellular function, and obstruction of portal circulation [3, 4]. There are many types of liver cirrhosis, such as cryptogenic cirrhosis, alcoholic liver cirrhosis, viral liver cirrhosis and NAFLD-associated cirrhosis, which are considered as the key risk factors for the occurrence of HCC [3–8]. Various polymorphisms of genes, such as patatin-like phospholipase domain containing 3 (PNPLA3), transmembrane 6 superfamily member (TM6SF2) 2 and methylenetetrahydrofolate reductase (MTHFR), are involved in susceptibility to the above liver diseases [9–16].

Human hereditary hemochromatosis (HFE) gene, first identified by Feder JN et al. in 1996, is located on the short arm of chromosome 6 (6p21.3) [17]. The HFE gene encodes a 343-amino acid glycoprotein (HFE protein), a member of the major histocompatibility complex class I-like family [17, 18]. As a key component of iron homeostasis in humans, the HFE protein is linked to the incidence of hereditary hemochromatosis (HH), an autosomal recessive disorder [17, 18]. Several common polymorphisms of the HFE gene, such as C282Y (rs1800562), H63D (rs1799945) and S65C (rs1800730), have been reported [18, 19]. Accumulating evidence indicates that HFE mutations are associated with susceptibility to many clinical diseases, such as Parkinson's disease (PD) [20], primary varicose veins [21] and coronary heart disease (CHD) [22].

Although several previous meta-analyses on the association of HFE genetic variants and NAFLD and HCC risk have been reported [12, 13, 23–26], a meta-analysis of the association of HFE gene mutation and overall liver cirrhosis has not been published, and more comprehensive systematic review and updated meta-analysis is therefore necessary to determine the relationship between HFE polymorphism and susceptibility to NAFLD, liver cirrhosis and HCC. Due to the limited data on S65C, we assessed the genetic risk conferred by the two common polymorphisms of HFE (C282Y and H63D). Our findings demonstrated that there is an association between HFE C282Y polymorphism and increased risk of NAFLD in the Caucasian population and HCC but not liver cirrhosis. Additionally, H63D polymorphism is likely to increase susceptibility to HCC without cirrhosis.

## Methods

The current meta-analysis followed the guidelines [27] of “Preferred Reporting Items for Systematic Reviews and Meta-Analyses (PRISMA)” and “Meta-analysis on Genetic Association Studies” (shown in S1 and S2 Tables) with small modifications.

### Systematic literature search

Five electronic databases (published prior to August 1^st^, 2016), including PubMed, EMBASE, Web of Science (WOS), Scopus and China National Knowledge Infrastructure (CNKI), were thoroughly searched for potential records. S3 Table presents the full details of the literature search based on the combination of index terms, such as “HFE”, “NAFLD”, “liver cirrhosis”, “HCC” and “single nucleotide polymorphism”. There were no restrictions of language, publication type or geographic location.

### Selection criteria and data extraction

Potential records from the systematic literature search were selected for eligible studies. We utilized EndNote X7 software to remove duplicate records and screened the title and abstract to exclude unrelated records based on the following criteria: (1) Review or book; (2) Not clinical data; (3) Other gene; (4) Other disease; (5) Case, trial, or non-polymorphism; (6) Meta-analysis; and (7) Meeting abstract. After the assessment of eligibility, all eligible articles were required to provide sufficient data regarding the genotype distribution of HFE polymorphism in both the case and control groups. The following basic information was extracted independently by the authors (QY BXQ WLY FMW): name of the first author, year of publication, country, ethnicity, sample sizes, source of control, genotyping methods for SNP, disease definition, diagnostic method, genotype frequencies, and the *P* value of the Hardy-Weinberg-Equilibrium (HWE) test in the control group. For unavailable or missing data, we attempted to contact the corresponding or first author through E-mail or the ResearchGate website.

### Quality assessment

Three authors (QY BXQ WLY) independently assessed the methodological quality of the included studies, according to the Newcastle-Ottawa Scale (NOS) system, which is available from http://www.ohri.ca/programs/clinicalepidemiology/oxford.html [28]. The NOS quality score system was used to critically evaluate the quality of non-randomized studies in the meta-analysis based on the following items: “case/control definition”, “representativeness of the cases”, “selection of controls”, “comparability of cases and controls” and “ascertainment of exposure”. An NOS score ≥ 7 was considered as a high-quality study. A thorough discussion with other authors was required to settle conflicting evaluations and discrepancies.

### Statistical analysis

The values of pooled odds ratios (ORs), 95% confidence intervals (CIs) and *P*_association_ were determined through Mantel-Haenszel statistics using Stata/SE 12.0 (Stata Corporation, TX, USA) software. A *P*_association_<0.05 indicated a significant difference between the case and control groups. Five inheritance models, namely, allele (Y vs C for C282Y, D vs H for H63D), homozygote (YY vs CC, DD vs HH), heterozygote (CY vs CC, HD vs HH), dominant (CY+YY vs CC, HD+DD vs HH) and recessive models (YY vs CC+CY, DD vs HH+HD), were applied.

Cochran’s Q statistic and I^2^ test were performed to evaluate the potential heterogeneities among studies. A random-effect model was used when the existence of significant heterogeneity was not excluded (*P*_Heterogeneity_ value of Q statistic<0.1 or I^2^ value>25%). To investigate the potential sources of heterogeneity, subgroup analyses based on ethnicity, source of controls, genotyping methods, HWE and disease type (such as NASH, alcoholic cirrhosis, cryptogenic cirrhosis) were conducted. In addition, both Begg’s test and Egger’s test were performed to assess the potential publication bias, and sensitivity analysis was conducted to evaluate whether the results were statistically stable. The *P*_HWE_ value was obtained from a chi-squared test, and *P*_HWE_ greater than 0.05 was considered as being in agreement with HWE.

## Results

### Characteristics of the eligible studies

There were 43 eligible articles with 5,758 cases and 14,741 controls included in our meta-analysis [7, 8, 10, 19, 29–67], including 16 articles on NAFLD [19, 29–43], 18 articles on liver cirrhosis [7, 8, 31, 44–58] and 17 articles on HCC [7, 10, 44, 46, 48, 51, 52, 58–67]. These articles met our inclusion/exclusion criteria. Thirty-nine studies of high quality (NOS score ≥6) [7, 8, 10, 29–36, 38–54, 56, 58–67] and 4 studies of moderate quality (NOS score = 6) [19, 37, 55, 57] were identified. S4 Table presents the summarized characteristics and methodological quality of selected studies, and S5 and S6 Tables show the genotype distributions of the HFE C282Y and H63D polymorphisms in NAFLD, liver cirrhosis and HCC disease.

**Fig 1:**
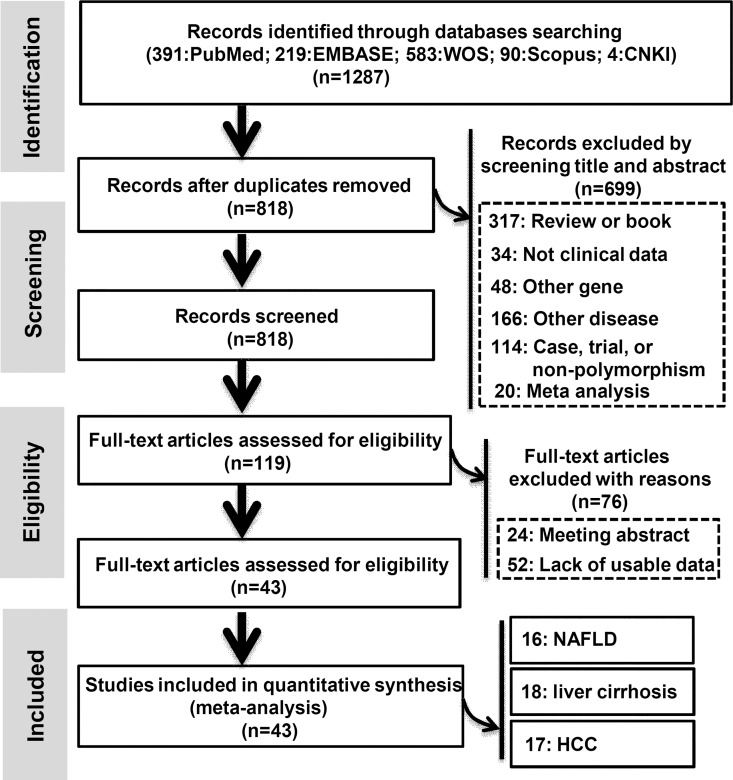
Flow diagram of the search strategy for the meta-analysis.

A total of 1,287 potential records were obtained from the PubMed (n = 391), EMBASE (n = 219), WOS (n = 583), Scopus (n = 90) and CNKI (n = 4) databases, and 469 duplicate records were removed by EndNote software. Additionally, 699 records were excluded by screening the title and abstract for the following: Review or book (n = 317), Not clinical data (n = 34), Other gene (n = 48), Other disease (n = 166); Case, trial, or non-polymorphism (n = 114); and Meta-analysis (n = 20). The authors (QY BXQ WLY FMW) independently extracted the data from 119 full-text articles and removed 76 articles, including 24 articles, which were meeting abstracts, and 52 articles, which lacked usable data. A flow diagram of the literature search strategy for the meta-analysis is presented in Fig 1.

### C282Y and H63D polymorphism and NAFLD risks

**Fig 2:**
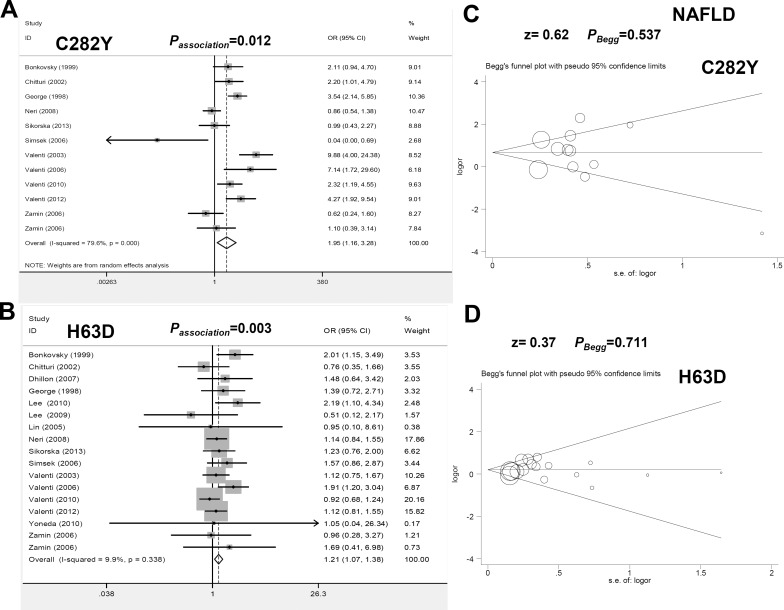
Meta-analysis of the genetic relationship between the C282Y and H63D polymorphisms of HFE and NAFLD risk under the allele model. (A) Forest plot for C282Y under the Y vs C model; (B) Forest plot for H63D under the D vs H model; (C) Begg’s test for C282Y; (D) Begg’s test for H63D.

We first investigated the genetic association between the HFE C282Y polymorphism and the susceptibility to NAFLD. A random-effect model was used for Mantel-Haenszel statistics due to the high degree of heterogeneity (Fig 2A and Table 1, all I^2^ >25%, *P*_heterogeneity_<0.1). The pooled results in Fig 2A and Table 1 show that compared with the control group, increased NAFLD risk was observed in the case group under the allele (OR = 1.95, *P*_*association*_ = 0.012), heterozygote (OR = 1.87, *P*_*association*_ = 0.016) and dominant models (OR = 1.95, *P*_*association*_ = 0.014) but not in the other models.

**Table 1:** Pooled analysis for the association between HFE C282Y, H63D genotype frequencies and the risks of NAFLD, liver cirrhosis, HCC.

				Test of association		Heterogeneity			Begg’s test		Egger’s test	
Disease	Site	Comparison	Number of studies	OR (95% CI)	*P*_*association*_	I^2^	*P*_heterogeneity_	Model	z	*P*_*Begg*_	t	*P*_*Egger*_
**NAFLD**	**C282Y**	Y vs C	12	1.95(1.16~3.28)	**0.012**	79.6	<0.001	R	0.62	0.537	-0.20	0.842
		YY vs CC	8	3.32(0.72~15.36)	0.125	63.8	0.007	R	-0.12	1.000	-1.65	0.151
		CY vs CC	12	1.87(1.12~3.12)	**0.016**	74.1	<0.001	R	0.48	0.631	0.15	0.881
		CY+YY vs CC	12	1.95(1.14~3.31)	**0.014**	77.1	<0.001	R	0.48	0.631	-0.05	0.957
		YY vs CC+CY	8	3.17(0.77~13.12)	0.112	57.9	0.020	R	-0.12	1.000	-1.62	0.156
	**H63D**	D vs H	17	1.21(1.07~1.38)	**0.003**	9.9	0.338	F	0.37	0.711	0.63	0.537
		DD vs HH	13	1.47(0.97~2.22)	0.069	0.0	0.895	F	1.28	0.200	1.67	0.124
		HD vs HH	17	1.22(1.05~1.41)	**0.010**	22.1	0.179	F	0.12	0.902	0.29	0.778
		HD+DD vs HH	17	1.24(1.07~1.43)	**0.004**	16.8	0.257	F	0.04	0.967	0.46	0.650
		DD vs HH+HD	13	1.35(0.90~2.03)	0.146	0.0	0.916	F	1.34	0.180	1.53	0.153
**liver cirrhosis**	**C282Y**	Y vs C	22	0.93(0.75~1.17)	0.536	0.0	0.727	F	0.51	0.612	0.21	0.834
		YY vs CC	9	0.88(0.41~1.91)	0.751	12.3	0.332	F	0.94	0.348	1.90	0.100
		CY vs CC	22	0.95(0.74~1.21)	0.671	0.0	0.924	F	0.45	0.652	-0.43	0.669
		CY+YY vs CC	22	0.94(0.74~1.19)	0.594	0.0	0.878	F	0.11	0.910	-0.10	0.923
		YY vs CC+CY	9	0.87(0.40~1.88)	0.728	9.2	0.358	F	1.15	0.251	1.93	0.095
	**H63D**	D vs H	31	0.68(0.60~0.76)	0.093	36.0	0.025	R	0.51	0.610	0.58	0.567
		DD vs HH	23	1.07(0.72~1.58)	0.743	0.0	0.506	F	2.43	**0.015**	1.98	0.061
		HD vs HH	31	1.18(0.98~1.41)	0.073	34.9	0.031	R	0.37	0.708	-0.05	0.962
		HD+DD vs HH	31	1.17(0.98~1.39)	0.082	35.1	0.030	R	0.14	0.892	0.25	0.801
		DD vs HH+HD	23	1.06(0.72~1.56)	0.777	0.0	0.499	F	2.48	**0.013**	1.72	0.100
**HCC**	**C282Y**	Y vs C	24	1.55(1.12~2.14)	**0.008**	52.6	0.001	R	0.82	0.413	-0.97	0.340
		YY vs CC	10	3.16(1.02~9.79)	**0.046**	57.8	0.011	R	1.07	0.283	-0.73	0.486
		CY vs CC	24	1.42(1.03~1.97)	**0.034**	42.4	0.016	R	0.57	0.568	-0.08	0.936
		CY+YY vs CC	24	1.51(1.09~2.10)	**0.013**	47.6	0.005	R	0.72	0.472	-0.54	0.592
		YY vs CC+CY	10	3.12(1.03~9.46)	**0.045**	56.4	0.014	R	1.07	0.283	-0.74	0.482
	**H63D**	D vs H	25	1.08(0.90~1.29)	0.436	44.5	0.009	R	0.30	0.761	-0.94	0.359
		DD vs HH	18	0.99(0.65~1.51)	0.954	23.0	0.182	F	2.05	**0.041**	-2.10	0.052
		HD vs HH	25	1.16(0.96~1.41)	0.122	32.4	0.061	R	0.02	0.981	-0.81	0.429
		HD+DD vs HH	25	1.13(0.92~1.38)	0.238	39.5	0.023	R	0.16	0.870	-0.84	0.412
		DD vs HH+HD	18	0.90(0.59~1.37)	0.626	16.3	0.258	F	2.35	**0.019**	-2.05	0.057

NAFLD: non-alcoholic fatty liver disease; HCC, hepatocellular carcinoma.

Moreover, subgroup analyses under all genetic models were conducted based on ethnicity (Asian, Caucasian and Mixed), source of controls (PB and HB), genotyping methods (PCR-RFLP and other), HWE (*P*_*HWE*_>0.05 and *P*_*HWE*_ <0.05) and specific disease type (NASH). As shown in S7 Table, a significantly increased NAFLD risk was observed in the Caucasian population, with *P*_*HWE*_ >0.05 subgroup (all OR>1, *P*_*association*_<0.05). These data suggested that HFE C282Y may be linked to the risk of NAFLD, especially in the Caucasian population.

An association between HFE H63D and NAFLD risk was also detected. No large heterogeneity was detected (Fig 2B and Table 1, all I^2^<25%, *P*_heterogeneity_>0.1). As shown in Fig 2B and Table 1, increased NAFLD risk was observed in the models of D vs H (OR = 1.21, *P*_*association*_ = 0.003), HD vs HH (OR = 1.22, *P*_*association*_ = 0.010), and HD+DD vs HH (OR = 1.24, *P*_*association*_ = 0.004). A similar significant difference was observed in the subgroup analysis for the Asian population, PB, PCR-RFLP, and NASH (S8 Table, *P*_*association*_<0.05, OR>1). Therefore, the HD genotype of HFE H63D contributes to increased NAFLD susceptibility.

### C282Y and H63D polymorphism and liver cirrhosis risk

**Fig 3:**
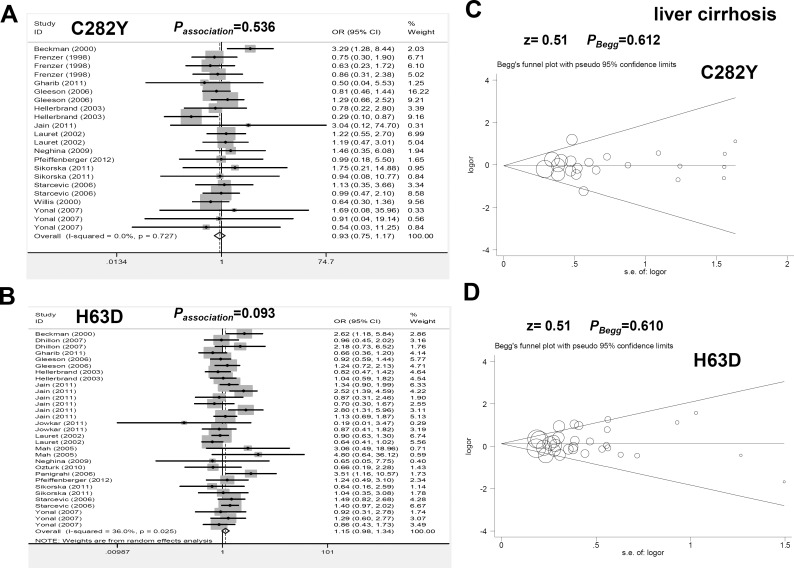
Meta-analysis of the genetic relationship between the C282Y and H63D polymorphisms of HFE and liver cirrhosis risk under the allele model. (A) Forest plot for C282Y under the Y vs C model; (B) Forest plot for H63D under the D vs H model; (C) Begg’s test for C282Y; (D) Begg’s test for H63D.

The data in Fig 3A and Table 1 show that a fixed-effect model was used for the meta-analysis of the association between HFE C282Y and liver cirrhosis risk (all I^2^<25%, *P*_heterogeneity_>0.1). No significant difference between the control and case group was observed for C282Y under all genetic models (Fig 3A, Table 1 and S7 Table, all *P*_*association*_>0.05). For H63D, as shown in Fig 3B and Table 1, a fixed-effect model was used for the homozygote and recessive contrasts (all I^2^ = 0.0%, *P*_heterogeneity_>0.1), whereas a random-effect model was used for the others (all I^2^>25.0%, *P*_heterogeneity_<0.1). There were no significant differences under the majority of comparisons in the meta-analysis and subsequent subgroup analysis (Table 1 and S8 Table, *P*_*association*_>0.05), except for the allele, homozygote and recessive models in the Asian population and the allele model in the *P*_HWE_>0.05 group. These data failed to provide strong evidence of a significant correlation between HFE C282Y and H63D and liver cirrhosis risk.

### C282Y and H63D polymorphism and HCC risk

**Fig 4:**
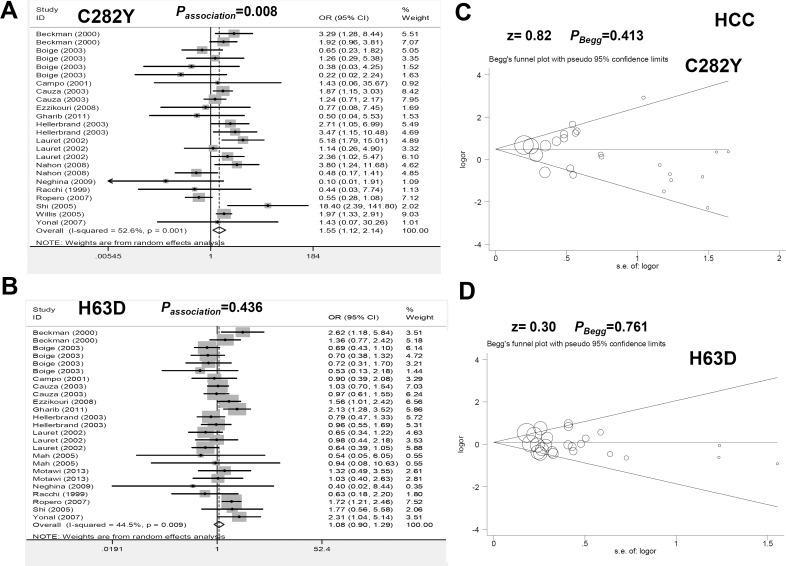
Meta-analysis of the genetic relationship between the C282Y and H63D polymorphisms of HFE and HCC risk under the allele model. (A) Forest plot for C282Y under the Y vs C model; (B) Forest plot for H63D under the D vs H model; (C) Begg’s test for C282Y; (D) Begg’s test for H63D.

A random-effect model was used to analyze the genetic association between HFE C282Y and HCC risk (Fig 4A and Table 1, all I^2^>25%, *P*_heterogeneity_<0.1), and an increased HCC risk was observed under all genetic models in the overall population (all OR>1, *P*_*association*_<0.05). A similar significant difference was observed in the pooled analysis in the PB, *P*_*HWE*_>0.05 and cirrhosis (-) subgroups under the allele, homozygote, dominant and recessive models (S7 Table, OR>1, *P*_*association*_<0.05). For H63D polymorphism, no increased HCC risk was observed in the overall population (Fig 4B and Table 1, all *P*_*association*_>0.05); however, there was a significant difference in the African population and the cirrhosis (-) subgroup (S8 Table, all OR>1, *P*_*association*_<0.05). Our data demonstrated that HFE C282Y may increase the odds of developing HCC, while HFE H63D is more likely associated with susceptibility to HCC without cirrhosis in the African population.

### Compound heterozygosity for C282Y/H63D and the risks of NAFLD, liver cirrhosis, and HCC

Next, to study the potential role of compound heterozygosity for C282Y/H63D in susceptibility to NAFLD, liver cirrhosis and HCC, we performed a meta-analysis and subgroup analysis. As shown in S1A–S3A Figs and S9 Table, a random-effect model was used for NAFLD, whereas fixed-effect models were used for liver cirrhosis and HCC. A significant difference was observed for NAFLD in the Caucasian population (OR = 2.13, *P*_*association*_ = 0.023) and for HCC in the overall population (OR = 1.70, *P*_*association*_ = 0.039) but not for liver cirrhosis (all *P*_*association*_>0.05). These data suggested that the effect of C282Y+H63D compound heterozygosity may contribute to an increased risk of NAFLD and HCC.

### Publication bias and sensitivity analysis

**Fig 5:**
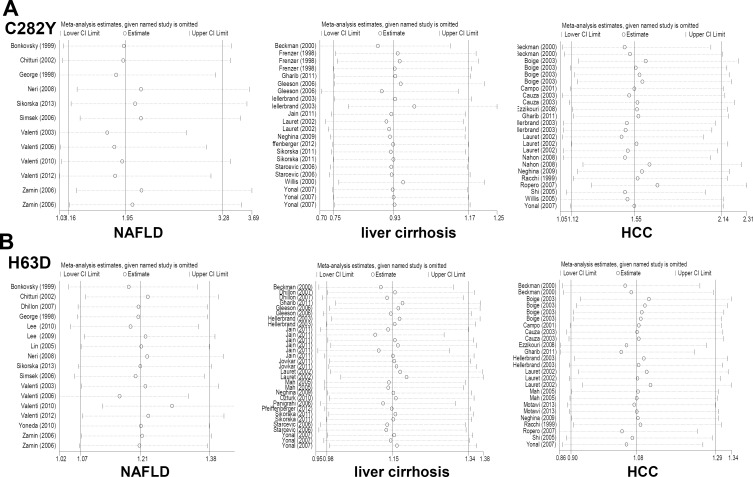
Sensitivity analysis of the association between the C282Y and H63D polymorphisms of HFE and the risks of diseases, including NAFLD, liver cirrhosis and HCC. (A) Y vs C model for C282Y; (B) D vs H model for H63D.

To evaluate the potential publication bias among the included studies, Begg’s test and Egger’s test were performed. For C282Y polymorphism, large publication bias was excluded under all genetic models for NAFLD, liver cirrhosis and HCC (Table 1 and Figs 2C, 3C and 4C, all *P*_Begg_>0.05, *P*_Eegger_>0.05). For H63D polymorphism, small publication bias was observed in the DD vs HH (Table 1, *P*_Begg_ = 0.015 for liver cirrhosis, *P*_Begg_ = 0.041 for HCC) and DD vs HH+HD models (Table 1, *P*_Begg_ = 0.013 for liver cirrhosis, *P*_Begg_ = 0.019 for HCC). However, there was no evidence of publication bias in the other models (Table 1 and Figs 2D, 3D and 4D, all *P*_Begg_>0.05, *P*_Eegger_>0.05). No obvious publication bias was observed for compound heterozygosity for C282Y/H63D (S1B, S1C, S3B and S3C Figs and S9 Table, all *P*_Begg_>0.05, *P*_Eegger_>0.05). Moreover, the sensitivity analysis further confirmed the statistical stability of our results (Fig 5 for the allele model; data not shown for the other models; S1D–S3D Figs for the C282Y+H63D mutation).

## Discussion

Evidence was obtained from several relevant genome-wide association studies (GWAS) [16, 68–70]. For instance, in 2010, Chalasani N, et al. performed GWAS to investigate the potential role of 324,623 allelic variants in 236 white women with NAFLD [69]. In 2013, Adams LA, et al. conducted another GWAS for NAFLD in 928-adolescents cohort [70]. The rs738409 polymorphism of PNPLA3 was identified in the GWAS of NAFLD [16]. However, the HFE gene was not included. In spite of this, polymorphisms of the HFE gene have been reported to be associated with NAFLD risk. For example, HFE H63D may contribute to the risk of NAFLD in the Korean population [33]. A positive association between HFE C282Y and NAFLD susceptibility was also observed [38]. Nevertheless, the role of HFE polymorphism in the occurrence of NAFLD remains unclear. For instance, there is no association between H63D of HFE and NASH risk in Italian patients [36].

In 2007, Ellervik C and colleagues reported that the HFE C282Y mutation might be linked to an increased risk of NASH under a homozygote meta-analysis model [23]. In 2011, Hernaez R, et al. performed a meta-analysis and failed to observe a positive association between HFE polymorphism and NAFLD susceptibility [12]. However, the results of our updated meta-analysis revealed that the HD genotype of H63D is more likely to be associated with NAFLD risk, and C282Y polymorphism may confer increased risk of NAFLD in the Caucasian population, which is partly consistent with the C282Y data of Ellervik C, et al. [23] but not Hernaez R, et al. [12]. How can this discrepancy be explained? The follow points were considered: (1) The two articles published by Sikorska K, et al. [19] and Valenti L, et al. [41] after 2011 and an additional three previously undetected articles [31, 34, 38] were added to our meta-analysis. In addition to a population-based control, the data of a hospital-based control were added to enhance the statistical power. For example, 20 patients with normal liver function tests and 20 patients infected with hepatitis C virus were considered as control groups based on the data of Zamin I, et al. [43]. (2) Sixteen case-control studies and 14 case-only studies were enrolled in the meta-analysis of Hernaez R, et al. [12]. Here, we utilized different evaluation criteria and focused on case-control studies that provided the data about the genotype distribution. The *P*_association_ value, OR and 95% CI were calculated using Mantel-Haenszel statistics under the allele, homozygote, heterozygote, dominant and recessive models. (3) NAFLD is not a uniform clinical disease entity [5, 6, 71], and different case definitions and pathological diagnoses are probably the most important source of heterogeneity. We therefore extracted the data of the case features and performed subgroup analysis on the basis of specific NAFLD types. Unfortunately, we only extracted sufficient data for meta-analysis of NASH but not NAFLD-associated fatty liver, liver cirrhosis or cirrhosis. No remarkable association between HFE C282Y and NASH risk or reduced heterogeneity (data no shown) were observed in our further subgroup analysis for NASH under all genetic models. However, we found a positive association between the HD genotype of HFE H63D and increased susceptibility to overall NAFLD and specific NASH, particularly in the Asian population.

Progressive iron overload in the liver was considered a key factor for the presence of liver injury, chronic inflammation, fibrosis, cirrhosis, liver failure and cancer [72, 73]. The C282Y and H63D polymorphisms of the HFE gene were tightly associated with the presence of HH with impaired iron metabolism [18, 19]. Mild iron overload was associated with HFE C282Y mutation and NAFLD risk [38]. The HFE protein was reported to be capable of forming a stable complex with transferrin receptor (TFR) to inhibit the abnormal up-regulation of the level of iron in cells, whereas the HFE C282Y mutation impairs the process and leads to peripheral iron overload [44, 74, 75]. In our meta-analysis, we observed an association between HFE C282Y and H63D and NAFLD risk. Iron overload might contribute to this association by acting as a fundamental regulation factor.

Diverse conclusions on the role of HFE polymorphism in HCC risk have also been obtained. For instance, the C282Y heterozygous genotype was reported to be associated with susceptibility to HCC [48]. H63D was linked to increased HCC risk in the Moroccan population [62]. However, the data of Racchi O, et al. showed that the HFE gene polymorphisms failed to participate in the pathogenesis of HCC [64]. Several related meta-analyses have been conducted [13, 24–26]. In 2010, Jin F, et al. reported an association between HCC susceptibility and C282Y, but not H63D, mutation in the European population [24]. Very recently, the meta-analysis of Lv YF, et al. showed that HFE C282Y mutation may be associated with increased HCC risk [25]. Additionally, another meta-analysis showed a positive association between HCC susceptibility and the YY homozygote genotype of C282Y but not the DD and HD genotypes of H63D [26]. Nevertheless, in 2015, Shen LL, et al. found that HFE H63D polymorphism might be involved in the aggressiveness of HCC [13]. In addition, conflicting data on the correlation between overall liver cirrhosis and HFE gene mutations were observed. For instance, the polymorphisms of the HFE gene are not essential for cryptogenic cirrhosis in the southern Iranian population [50]. C282Y might be linked to the risk of HCC in patients with alcoholic-related cirrhosis [24, 63]. H63D was reported to be associated with increased HCC risk in cirrhotic patients [65]. However, Boige V, et al. reported that the C282Y and H63D polymorphisms were not associated with increased susceptibility to HCC plus cirrhosis in patients [59]. Therefore, we performed an updated meta-analysis to better understand the genetic relationship between HFE mutations and the risks of HCC and liver cirrhosis. Our data demonstrated that C282Y and H63D are not associated with the risks of alcoholic, cryptogenic or viral-related liver cirrhosis. Moreover, HFE C282Y was significantly linked to the risk of HCC, while H63D was more likely to be involved in susceptibility to HCC without cirrhosis.

The disadvantages of our systematic review and meta-analysis are as follows. (1) The eligible articles contain relatively small sample sizes. For instance, only two case-control studies were enrolled in the meta-analysis of the Asian subgroup. (2) Four moderate-quality studies were included in our meta-analysis [19, 37, 55, 57]. The lack of sufficient information for the “case definition” or “representativeness of the cases” and the selection of a non-community control might contribute to this quality issue. (3) Additional unpublished articles, between-study heterogeneity and potential publication bias may distort our conclusions. (4) There are highly various etiologies for NAFLD, liver cirrhosis and HCC [3, 4, 6]. We failed to obtain efficient phenotype data and thus performed very limited stratified meta-analyses, which might contribute to the heterogeneity among studies. Additional well-powered studies are required to confirm the effect of multiple HFE mutations (C282Y, H63D and S65C) on the susceptibility to different types of NAFLD, liver cirrhosis and HCC.

## Conclusion

In summary, our updated systematic review and meta-analysis confirmed the role of HFE C282Y in an increased HCC risk and provided new evidence that H63D is more likely to be associated with susceptibility to non-cirrhotic HCC in the African population. A significant correlation between HFE C282Y and H63D polymorphism and NAFLD susceptibility was obtained. Furthermore, we found that the HFE mutations failed to increase the odds of developing liver cirrhosis. The first evidence regarding the positive genetic relationship between compound heterozygosity for C282Y/H63D and the risks of NAFLD and HCC was demonstrated.

## Supporting Information

S1 Fig Meta-analysis of the genetic relationship between C282Y+H63D polymorphism and NAFLD risk. (A) Forest plot analysis; (B) Begg’s test; (C) Egger’s test; (D) Sensitivity analysis.
(TIF)

S2 Fig Meta-analysis of the genetic relationship between C282Y+H63D polymorphism and liver cirrhosis risk. (A) Forest plot analysis; (B) Begg’s test; (C) Egger’s test; (D) Sensitivity analysis.
(TIF)

S3 Fig Meta-analysis of the genetic relationship between C282Y+H63D polymorphism and HCC risk. (A) Forest plot analysis; (B) Begg’s test; (C) Egger’s test; (D) Sensitivity analysis.
(TIF)

S1 Table PRISMA 2009 checklist. (DOCX)

S2 Table Meta-analysis on genetic association studies checklist. (DOCX)

S3 Table Electronic databases searching terms for meta-analysis. (DOCX)

S4 Table Characteristics of studies included in the meta-analysis. (DOCX)

S5 Table Genotype distribution of HFE C282Y polymorphism. (DOCX)

S6 Table Genotype distribution of HFE H63D polymorphism. (DOCX)

S7 Table Subgroup analyses for HFE C282Y. (DOCX)

S8 Table Subgroup analyses for HFE H63D. (DOCX)

S9 Table Pooled analysis of the association between the HFE C282Y+H63D genotype frequencies and the risks of NAFLD, liver cirrhosis, and HCC. (DOCX)
