# Supplementary figures and images for: Association between the HFE C282Y, H63D Polymorphisms and the Risks of Non-Alcoholic Fatty Liver Disease, Liver Cirrhosis and Hepatocellular Carcinoma: An Updated Systematic Review and Meta-Analysis of 5,758 Cases and 14,741 Controls
Source: PLoS One. 2016 Sep 22;11(9):e0163423. doi: 10.1371/journal.pone.0163423 (PMC5033482; doi:10.1371/journal.pone.0163423)

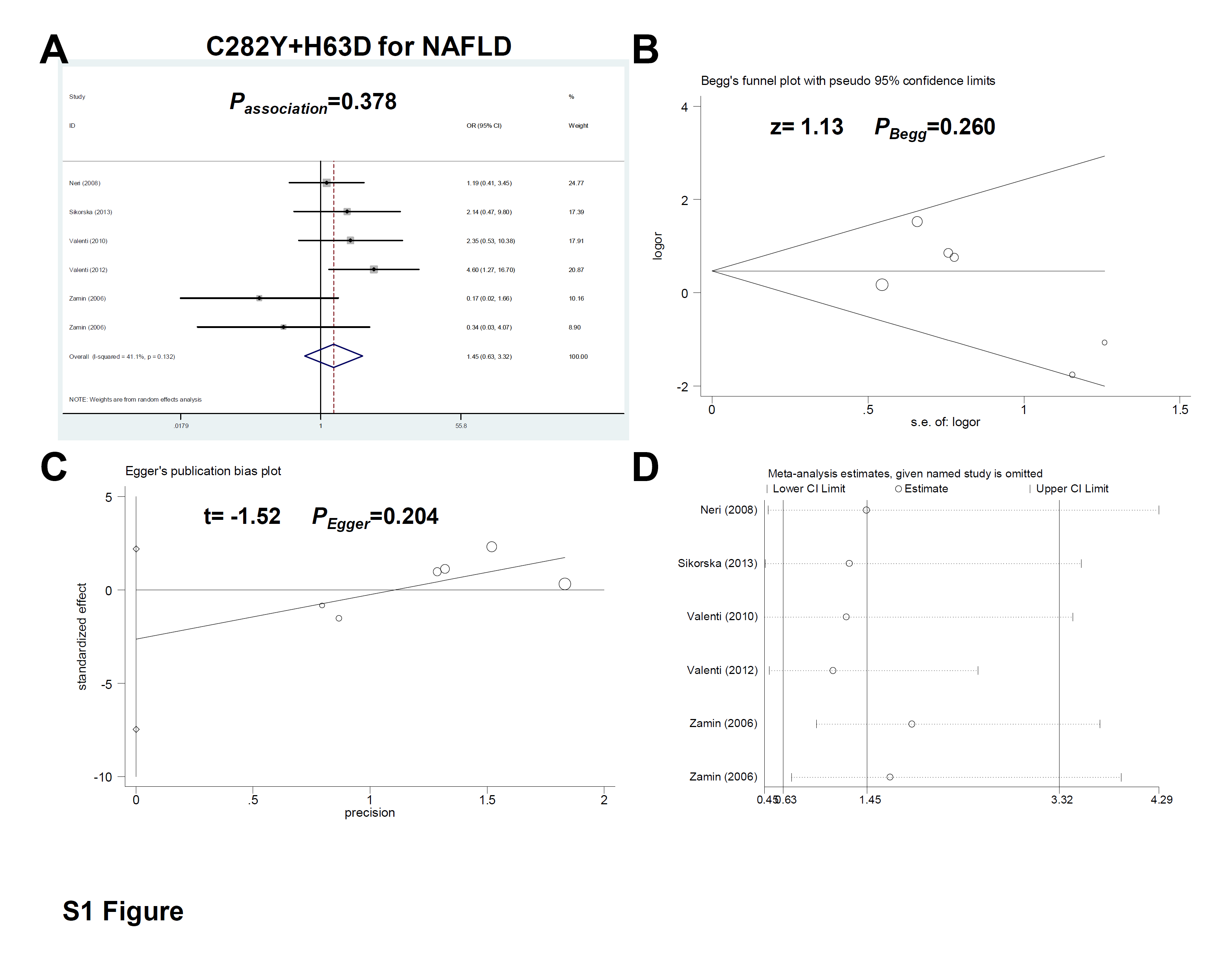

Supplement: S1 Fig — (A) Forest plot analysis; (B) Begg’s test; (C) Egger’s test; (D) Sensitivity analysis. (TIF) [file pone.0163423.s001.tif]

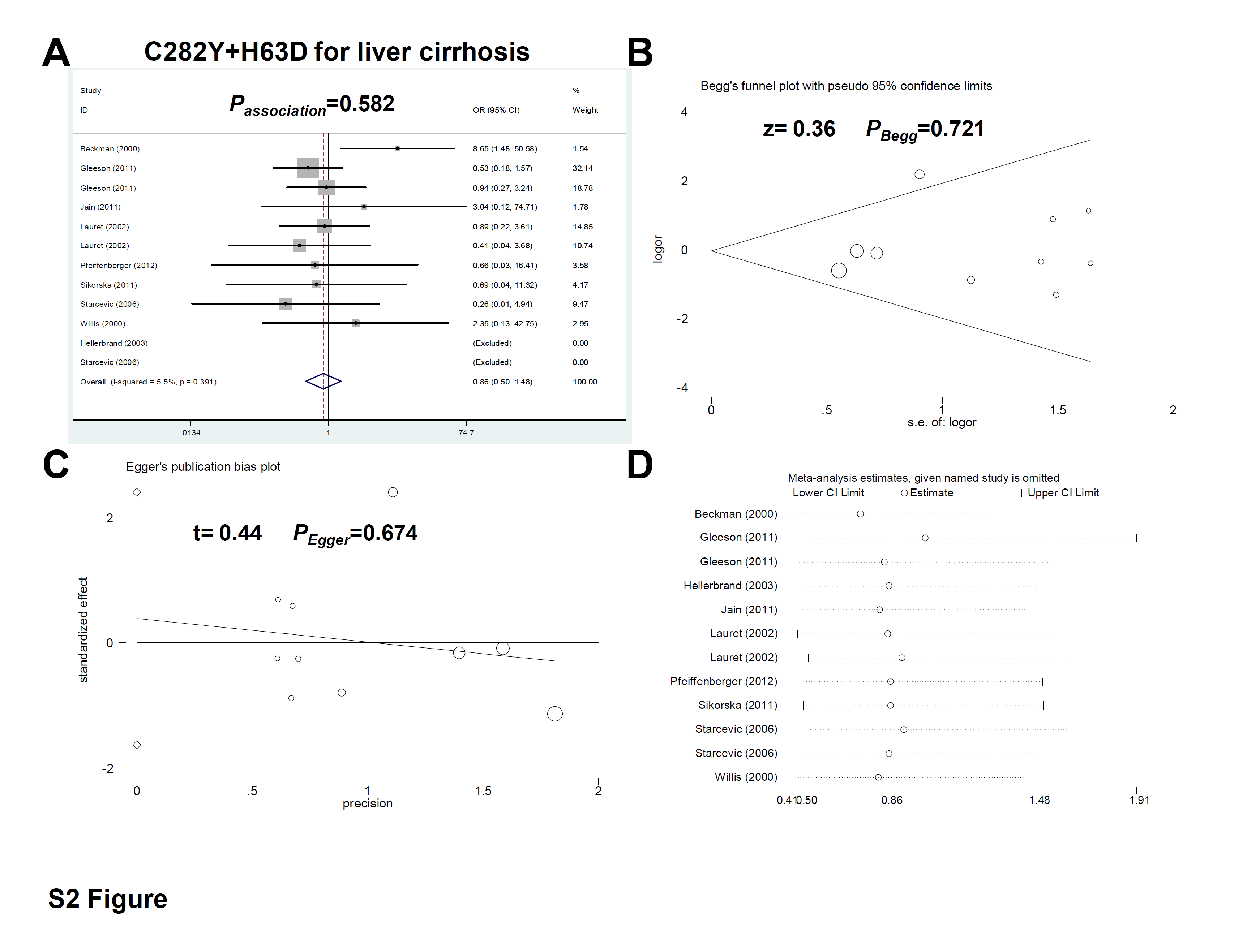

Supplement: S2 Fig — (A) Forest plot analysis; (B) Begg’s test; (C) Egger’s test; (D) Sensitivity analysis. (TIF) [file pone.0163423.s002.tif]

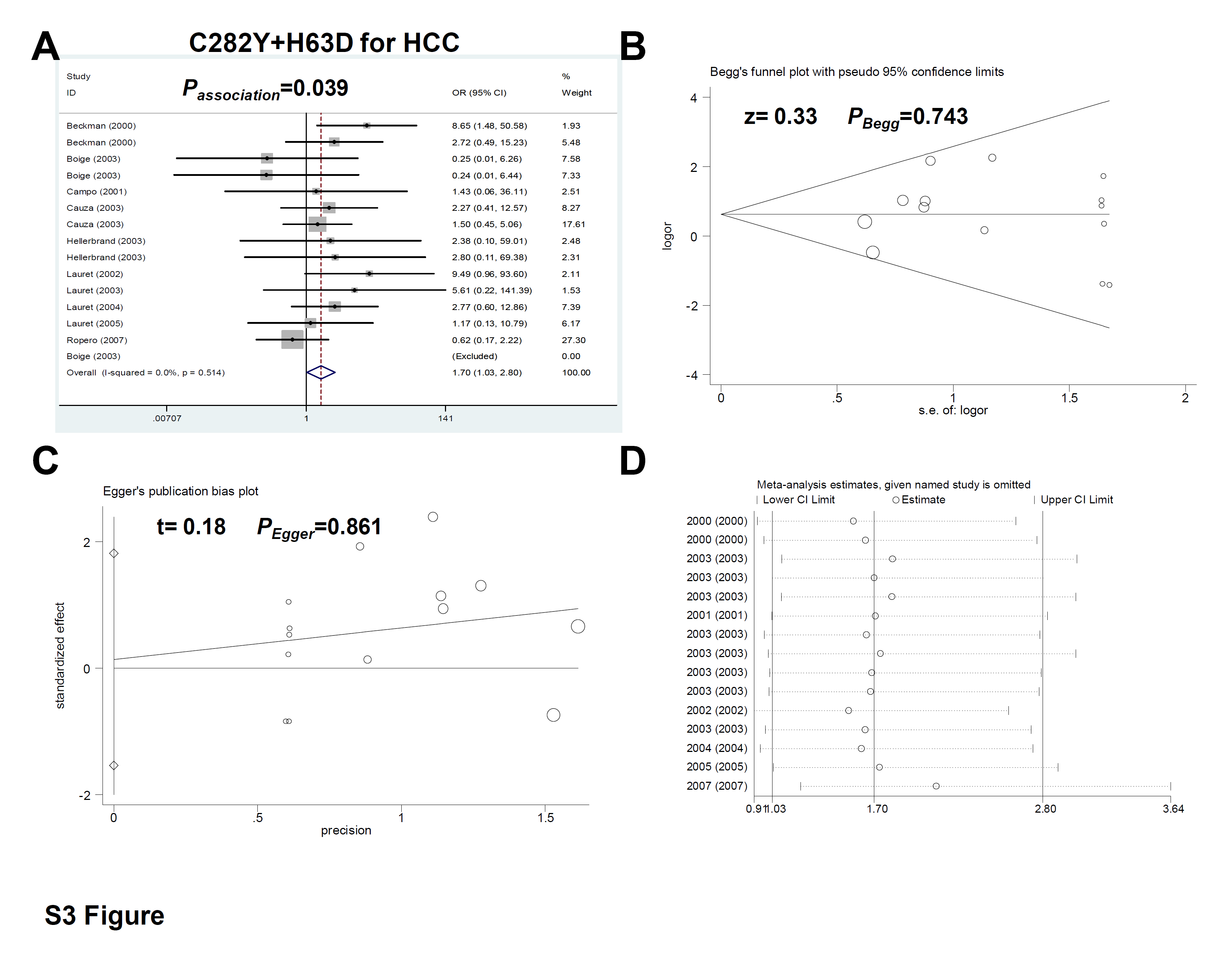

Supplement: S3 Fig — (A) Forest plot analysis; (B) Begg’s test; (C) Egger’s test; (D) Sensitivity analysis. (TIF) [file pone.0163423.s003.tif]
