# Supplementary material for: Association between the HFE C282Y, H63D Polymorphisms and the Risks of Non-Alcoholic Fatty Liver Disease, Liver Cirrhosis and Hepatocellular Carcinoma: An Updated Systematic Review and Meta-Analysis of 5,758 Cases and 14,741 Controls
Source: PLoS One. 2016 Sep 22;11(9):e0163423. doi: 10.1371/journal.pone.0163423 (PMC5033482; doi:10.1371/journal.pone.0163423)
Supplement: S3 Table — (DOCX) [file pone.0163423.s006.docx]

# S3 Table Electronic databases searching terms for meta-analysis.

| Database* | Searching Terms | Number of articles |
| --- | --- | --- |
| Pubmed | ((((((((Liver Cirrhosis) OR Non-alcoholic Fatty Liver Disease) OR NAFLD) OR Nonalcoholic Steatohepatitis) OR Hepatocellular Carcinoma) OR HCC)) AND ((((((((polymorphism) OR polymorphisms) OR variant) OR variants) OR mutation) OR mutations) OR SNP) OR Single Nucleotide Polymorphism)) AND ((hemochromatosis gene) OR HFE) | 391 |
| EMBASE | ('hemochromatosis gene' OR 'HFE') AND ('nonalcoholic fatty liver' OR 'NAFLD' OR 'Nonalcoholic Steatohepatitis' OR 'Hepatocellular Carcinoma' OR 'HCC') AND ('polymorphism' OR 'polymorphisms' OR 'variant' OR 'variants' OR 'mutation' OR 'mutations' OR 'SNP' OR 'Single Nucleotide Polymorphism') AND [humans]/lim | 219 |
| WOS | TOPIC:(hemochromatosis gene OR HFE) AND TOPIC: (polymorphism OR polymorphisms OR variant OR variants OR mutation OR mutations OR SNP OR Single Nucleotide Polymorphism) AND TOPIC: (Liver Cirrhosis OR Non-alcoholic Fatty Liver Disease OR NAFLD OR Nonalcoholic Steatohepatitis OR Hepatocellular Carcinoma OR HCC) Timespan: All years. Search language=Auto | 583 |
| Scopus | ('hemochromatosis gene' OR 'HFE') AND ('nonalcoholic fatty liver' OR 'NAFLD' OR 'Nonalcoholic Steatohepatitis' OR 'Hepatocellular Carcinoma' OR 'HCC') AND ('polymorphism' OR 'polymorphisms' OR 'variant' OR 'variants' OR 'mutation' OR 'mutations' OR 'SNP' OR 'Single Nucleotide Polymorphism') | 90 |
| CNKI | HFE 基因多态性 非酒精性脂肪肝 肝硬化 肝细胞癌 | 4 |

* updated to August 1^st^, 2016
