# Supplementary material for: Association between the HFE C282Y, H63D Polymorphisms and the Risks of Non-Alcoholic Fatty Liver Disease, Liver Cirrhosis and Hepatocellular Carcinoma: An Updated Systematic Review and Meta-Analysis of 5,758 Cases and 14,741 Controls
Source: PLoS One. 2016 Sep 22;11(9):e0163423. doi: 10.1371/journal.pone.0163423 (PMC5033482; doi:10.1371/journal.pone.0163423)
Supplement: S4 Table — (DOCX) [file pone.0163423.s007.docx]

S4 Table Characteristics of studies included in the meta-analysis.

| First author | Year | Country | Ethnicity | Disease | Site | Case | Control | Source of control | Genotyping methods | NOS score |
| --- | --- | --- | --- | --- | --- | --- | --- | --- | --- | --- |
| Beckman | 2000 | Sweden | Caucasian | Cirrhosis | C282Y | 17 | 294 | PB | PCR-RFLP | 7 |
|  |  |  |  | Cirrhosis | H63D | 17 | 294 |  |  |  |
|  |  |  |  | Cirrhosis | C282Y+H63D | 2 | 4 |  |  |  |
|  |  |  |  | HCC | C282Y | 71 | 294 |  |  |  |
|  |  |  |  | HCC | H63D | 71 | 294 |  |  |  |
|  |  |  |  | Cirrhosis | C282Y+H63D | 4 | 8 |  |  |  |
| Boige | 2003 | France | Caucasian | HCC | C282Y | 133 | 100 | HB | PCR-RFLP | 7 |
|  |  |  |  | HCC | H63D | 133 | 100 |  |  |  |
|  |  |  |  | HCC | C282Y+H63D | 0 | 2 |  |  |  |
| Bonkovsky | 1999 | USA | Caucasian | NAFLD | C282Y | 36 | 348 | PB | PCR-RFLP | 8 |
|  |  |  |  | NAFLD | H63D | 36 | 348 |  |  |  |
| Campo | 2001 | Italy | Caucasian | HCC | C282Y | 23 | 100 | PB | PCR-RFLP | 8 |
|  |  |  |  | HCC | H63D | 23 | 100 |  |  |  |
|  |  |  |  | HCC | C282Y+H63D | 0 | 1 |  |  |  |
| Cauza | 2003 | Austria | Mixed | HCC | C282Y | 162 | 671 | PB+HB | PCR-RFLP | 7 |
|  |  |  |  | HCC | H63D | 162 | 671 |  |  |  |
|  |  |  |  | HCC | C282Y+H63D | 8 | 10 |  |  |  |
| Chitturi | 2002 | Australia | Caucasian | NAFLD | C282Y | 59 | 141 | PB | PCR-SSCP | 7 |
|  |  |  |  | NAFLD | H63D | 42 | 125 |  |  |  |
| Dhillon | 2007 | India | Asian | NAFLD | H63D | 59 | 100 | PB | PCR-RFLP | 8 |
|  |  |  |  | Cirrhosis | H63D | 155 | 100 |  |  |  |
| Ezzikouri | 2008 | Maroc | African | HCC | C282Y | 96 | 222 | PB+HB | PCR-RFLP | 7 |
|  |  |  |  | HCC | H63D | 96 | 222 |  |  |  |
| Frenzer | 1998 | Australia | Caucasian | Cirrhosis | C282Y | 61 | 157 | PB | PCR-RFLP | 7 |
|  |  |  |  | Cirrhosis | C282Y | 61 | 68 | HB |  |  |
| George | 1998 | Australia | Caucasian | NAFLD | C282Y | 51 | 2375 | PB | PCR-RFLP | 9 |
|  |  |  |  | NAFLD | H63D | 51 | 90 |  |  |  |
| Gharib | 2011 | Egypt | African | Cirrhosis | C282Y | 100 | 100 | PB | PCR-RFLP | 9 |
|  |  |  |  | Cirrhosis | H63D | 100 | 100 |  |  |  |
|  |  |  |  | HCC | C282Y | 100 | 100 |  |  |  |
|  |  |  |  | HCC | H63D | 100 | 100 |  |  |  |
| Gleeson | 2006 | Ireland, UK | Caucasian | Cirrhosis | C282Y | 252 | 130 | PB+HB | BIPASA | 7 |
|  |  |  |  | Cirrhosis | H63D | 252 | 130 |  |  |  |
|  |  |  |  | Cirrhosis | C282Y+H63D | 10 | 16 |  |  |  |
| Hellerbrand | 2003 | Germany | Caucasian | Cirrhosis | C282Y | 107 | 263 | PB+HB | PCR-RFLP | 7 |
|  |  |  |  | Cirrhosis | H63D | 107 | 263 |  |  |  |
|  |  |  |  | Cirrhosis | C282Y+H63D | 0 | 0 |  |  |  |
|  |  |  |  | HCC | C282Y | 137 | 233 |  |  |  |
|  |  |  |  | HCC | H63D | 137 | 233 |  |  |  |
|  |  |  |  | HCC | C282Y+H63D | 2 | 0 |  |  |  |
| Jain | 2011 | India | Asian | Cirrhosis | C282Y | 496 | 502 | PB | PCR-RFLP | 9 |
|  |  |  |  | Cirrhosis | H63D | 496 | 502 |  |  |  |
|  |  |  |  | Cirrhosis | C282Y+H63D | 1 | 0 |  |  |  |
| Jowkar | 2011 | Iran | Asian | Cirrhosis | H63D | 100 | 50 | PB | PCR-RFLP | 7 |
| Lauret | 2002 | Spain | Caucasian | Cirrhosis | C282Y | 277 | 159 | PB | PCR-RFLP | 7 |
|  |  |  |  | Cirrhosis | H63D | 277 | 159 |  |  |  |
|  |  |  |  | Cirrhosis | C282Y+H63D | 5 | 8 |  |  |  |
|  |  |  |  | HCC | C282Y | 154 | 359 | PB+HB |  |  |
|  |  |  |  | HCC | H63D | 154 | 359 |  |  |  |
|  |  |  |  | HCC | C282Y+H63D | 8 | 9 |  |  |  |
| Lee | 2009 | Korea | Asian | NAFLD | H63D | 43 | 441 | HB | PCR-RFLP | 7 |
| Lee | 2010 | Korea | Asian | NAFLD | H63D | 125 | 221 | PB | PCR-RFLP | 9 |
| Lin | 2005 | China | Asian | NAFLD | H63D | 33 | 125 | PB | PCR-RFLP | 9 |
| Mah | 2005 | China | Asian | Cirrhosis | H63D | 36 | 49 | PB | PCR-RFLP | 7 |
|  |  |  |  | HCC | H63D | 71 | 49 |  |  |  |
| Motawi | 2013 | Egypt | African | HCC | H63D | 39 | 80 | PB+HB | PCR-RFLP | 7 |
| Nahon | 2008 | France | Caucasian | HCC | C282Y | 103 | 198 | HB | TaqMan PCR, MGB probes | 7 |
| Neghina | 2009 | Norway | Caucasian | Cirrhosis | C282Y | 9 | 12 | HB | melting curve analysis with lightcycler | 7 |
|  |  |  |  | Cirrhosis | H63D | 9 | 12 |  |  |  |
|  |  |  |  | HCC | C282Y | 5 | 16 |  |  |  |
|  |  |  |  | HCC | H63D | 5 | 16 |  |  |  |
| Neri | 2008 | Italy | Caucasian | NAFLD | C282Y | 272 | 430 | PB | real-time ABI PRISM 7900, DNA sequencer | 8 |
|  |  |  |  | NAFLD | H63D | 272 | 430 |  |  |  |
|  |  |  |  | NAFLD | C282Y+H63D | 6 | 8 |  |  |  |
| Ozturk | 2010 | Turkey | Asian | Cirrhosis | H63D | 18 | 141 | PB |  |  |
| Panigrahi | 2006 | India | Asian | Cirrhosis | H63D | 31 | 74 | PB | PCR-RFLP | 9 |
| Pfeiffenberger | 2012 | Germany | Caucasian | Cirrhosis | C282Y | 48 | 95 | HB | multiplex dual-colour PCR | 7 |
|  |  |  |  | Cirrhosis | H63D | 48 | 95 |  |  |  |
|  |  |  |  | Cirrhosis | C282Y+H63D | 0 | 1 |  |  |  |
| Racchi | 1999 | Italy | Caucasian | HCC | C282Y | 12 | 130 | PB | PCR-fluorescent sequencing | 7 |
|  |  |  |  | HCC | H63D | 12 | 130 |  |  |  |
| Ropero | 2007 | Spain | Caucasian | HCC | C282Y | 196 | 181 | PB | PCR-RFLP | 8 |
|  |  |  |  | HCC | H63D | 196 | 181 |  |  |  |
|  |  |  |  | HCC | C282Y+H63D | 4 | 6 |  |  |  |
| Shi | 2005 | China | Asian | HCC | C282Y | 56 | 60 | PB | PCR-RFLP | 9 |
|  |  |  |  | HCC | H63D | 56 | 60 |  |  |  |
| Sikorska | 2011 | Poland | Caucasian | Cirrhosis | C282Y | 61 | 42 | HB | PCR-RFLP | 6 |
|  |  |  |  | Cirrhosis | H63D | 61 | 42 |  |  |  |
|  |  |  |  | Cirrhosis | C282Y+H63D | 1 | 1 |  |  |  |
| Sikorska | 2013 | Poland | Caucasian | NAFLD | C282Y | 67 | 191 | HB | PCR-RFLP | 6 |
|  |  |  |  | NAFLD | H63D | 67 | 191 |  |  |  |
|  |  |  |  | NAFLD | C282Y+H63D | 3 | 4 |  |  |  |
| Simsek | 2006 | Turkey | Asian | NAFLD | C282Y | 30 | 2677 | PB | PCR-RFLP | 6 |
|  |  |  |  | NAFLD | H63D | 30 | 2677 |  |  |  |
| Starcevic | 2006 | Croatia, Slovenia | Caucasian | Cirrhosis | C282Y | 147 | 416 | PB | PCR-RFLP | 7 |
|  |  |  |  | Cirrhosis | H63D | 147 | 416 |  |  |  |
|  |  |  |  | Cirrhosis | C282Y+H63D | 0 | 4 |  |  |  |
| Valenti | 2003 | Italy | Caucasian | NAFLD | C282Y | 134 | 291 | PB | PCR-RFLP | 8 |
|  |  |  |  | NAFLD | H63D | 134 | 291 |  |  |  |
| Valenti | 2006 | Italy | Caucasian | NAFLD | C282Y | 353 | 89 | PB | PCR-RFLP | 9 |
|  |  |  |  | NAFLD | H63D | 353 | 89 |  |  |  |
| Valenti | 2010 | Italy | Caucasian | NAFLD | C282Y | 599 | 184 | PB | PCR-RFLP | 9 |
|  |  |  |  | NAFLD | H63D | 587 | 184 |  |  |  |
|  |  |  |  | NAFLD | C282Y+H63D | 15 | 2 |  |  |  |
| Valenti | 2012 | Italy | Caucasian | NAFLD | C282Y | 216 | 271 | PB | PCR-SAS | 9 |
|  |  |  |  | NAFLD | H63D | 216 | 271 |  |  |  |
|  |  |  |  | NAFLD | C282Y+H63D | 11 | 3 |  |  |  |
| Willis | 2000 | England | Caucasian | Cirrhosis | C282Y | 190 | 34 | HB | PCR-RFLP | 6 |
|  |  |  |  | Cirrhosis | C282Y+H63D | 6 | 0 | HB |  |  |
| Willis | 2005 | England | Caucasian | HCC | C282Y | 144 | 1508 | PB | allelic discrimination-PCR | 7 |
| Yonal | 2007 | Turkey | Asian | Cirrhosis | C282Y | 97 | 138 | PB | PCR-RFLP | 7 |
|  |  |  |  | Cirrhosis | H63D | 97 | 138 |  |  |  |
|  |  |  |  | HCC | C282Y | 19 | 138 |  |  |  |
|  |  |  |  | HCC | H63D | 19 | 138 |  |  |  |
| Yoneda | 2010 | Japan | Asian | NAFLD | H63D | 58 | 20 | HB | PCR-RFLP | 7 |
| Zamin | 2006 | Brazil | Mixed | NAFLD | C282Y | 29 | 40 | HB | PCR-RFLP | 7 |
|  |  |  |  | NAFLD | H63D | 29 | 40 |  |  |  |
|  |  |  |  | NAFLD | C282Y+H63D | 2 | 6 |  |  |  |

PB: population-based; HB: Hospital-based; PCR-RFLP: polymerase chain reaction–restriction fragment length polymorphism; PCR-SSCP: Polymerase chain reaction-single strand conformation polymorphism; BIPASA: bi-directional PCR amlification of specific alleles; PCR-SAS: Polymerase chain reaction-sequence allele specific; NOS: Newcastle-Ottawa Scale.
