# Supplementary material for: Association between the HFE C282Y, H63D Polymorphisms and the Risks of Non-Alcoholic Fatty Liver Disease, Liver Cirrhosis and Hepatocellular Carcinoma: An Updated Systematic Review and Meta-Analysis of 5,758 Cases and 14,741 Controls
Source: PLoS One. 2016 Sep 22;11(9):e0163423. doi: 10.1371/journal.pone.0163423 (PMC5033482; doi:10.1371/journal.pone.0163423)
Supplement: S5 Table — (DOCX) [file pone.0163423.s008.docx]

S5 Table Genotype distribution of HFE C282Y polymorphism.

|  |  |  | Case | | |  |  | Control | | |  |
| --- | --- | --- | --- | --- | --- | --- | --- | --- | --- | --- | --- |
| Group | **First author** | **Year** | **CC** | **CY** | **YY** | **Disease** | **Diagnostic method or definition** | **CC** | **CY** | **YY** | ***P*_HWE_** |
| NAFLD | **Bonkovsky** | 1999 | 29 | 6 | 1 | NASH | clinical/laboratory examination and histopathological analysis | 309 | 39 | 0 | 0.27 |
|  | **Chitturi** | 2002 | 46 | 13 | 0 | NASH | based on the criteria of Brunt et al^&^ | 127 | 13 | 1 | 0.31 |
|  | **George** | 1998 | 35 | 12 | 4 | NASH | clinical/laboratory examination; liver biopsy; predominantly macrovesicular steatosis with lobular inflammation. | 2085 | 274 | 16 | **0.04** |
|  | **Neri** | 2008 | 244 | 28 | 0 | NASH | clinical/laboratory examination; ultrasonographic imaging; liver biopsy, | 379 | 51 | 0 | 0.19 |
|  | **Sikorska** | 2013 | 59 | 8 | 0 | NAFLD | clinical/laboratory examination; liver ultrasonographic imaging, liver biopsy; increased level of blood fatty acids without other liver damaging factors. | 171 | 17 | 3 | **<0.05** |
|  | **Simsek** | 2006 | 30 | 0 | 0 | NASH | not reported | 2013 | 458 | 206 | **<0.05** |
|  | **Valenti** | 2003 | 110 | 23 | 1 | NAFLD | clinical/laboratory examination; ultrasonographic imaging and liver biopsy | 285 | 6 | 0 | 0.86 |
|  | **Valenti** | 2006 | 307 | 39 | 7 | NAFLD | clinical/laboratory examination; liver biopsy; other causes of liver disease were excluded. | 87 | 2 | 0 | 0.91 |
|  | **Valenti** | 2010 | 538 | 49 | 12 | NAFLD | clinical/laboratory examination; liver biopsy; | 174 | 10 | 0 | 0.70 |
|  | **Valenti** | 2012 | 190 | 26 | 0 | NAFLD | clinical /laboratory examination; liver biopsy; | 263 | 8 | 0 | 0.81 |
|  | **Zamin** | 2006 | 18 | 11 | 0 | NASH | clinical/laboratory examination; liver biopsy | 9 | 11 | 0 | 0.09 |
|  |  |  | 18 | 11 | 0 | NASH |  | 13 | 7 | 0 | 0.34 |
| liver  cirrhosis | **Beckman** | 2000 | 12 | 4 | 1 | cirrhosis with HCC | liver biopsies and autopsies for hcc; and verified cirrhosis was defined as the disseminated occurrence of pseudolobuli surrounded by connective tissue. | 259 | 34 | 1 | 0.92 |
|  | **Frenzer** | 1998 | 54 | 7 | 0 | alcoholic cirrhosis | clinical /laboratory examination; ultrasonographic imaging and liver biopsy | 87 | 11 | 2 | **0.04** |
|  |  |  | 54 | 7 | 0 |  |  | 47 | 10 | 0 | 0.47 |
|  |  |  | 54 | 7 | 0 |  |  | 59 | 9 | 0 | 0.56 |
|  | **Gharib** | 2011 | 99 | 1 | 0 | HCV-related cirrhosis | clinical /laboratory examination; child-pugh score | 98 | 2 | 0 | 0.92 |
|  | **Gleeson** | 2006 | 157 | 24 | 2 | presumed alcoholic cirrhosis | clinical /laboratory examination; ultrasonographic imaging and liver biopsy | 108 | 20 | 2 | 0.35 |
|  |  |  | 54 | 14 | 1 | biopsy proven alcoholic cirrhosis |  | 108 | 20 | 2 | 0.35 |
|  | **Hellerbrand** | 2003 | 103 | 4 | 0 | mixed cirrhosis without HCC | clinical /laboratory examination; liver biopsy for complicated cirrhoisis without hcc, such as cryptogenic cirrhosis. | 120 | 6 | 0 | 0.78 |
|  |  |  | 103 | 4 | 0 |  |  | 120 | 17 | 0 | 0.44 |
|  | **Jain** | 2011 | 495 | 1 | 0 | mixed cirrhosis, including HBV/HCV related cirrhosis, cryptogenic cirrhosis; alcoholic cirrhosis with or without hepatitis | clinical/ laboratory examination; ultrasonographic imaging and liver biopsy for the diagnosis | 502 | 0 | 0 | - |
|  | **Lauret** | 2002 | 164 | 15 | 0 | alcoholic cirrhosis | clinical/ laboratory examination; ultrasonographic imaging and liver biopsy | 148 | 11 | 0 | 0.65 |
|  |  |  | 90 | 8 | 0 | HBV/HCV-related cirrhosis |  | 148 | 11 | 0 | 0.65 |
|  | **Neghina** | 2009 | 6 | 1 | 2 | mixed cirrhosis with/without HCC or HCV infection | clinical / laboratory examination | 9 | 1 | 2 | **0.01** |
|  | **Pfeiffenberger** | 2012 | 47 | 0 | 1 | cirrhosis with Wilson disease | clinical /laboratory examination and liver biopsy | 91 | 4 | 0 | 0.83 |
|  | **Sikorska** | 2011 | 36 | 5 | 2 | Mixed cirrhosis-elevated serum iron | clinical / laboratory examination and liver biopsy | 7 | 1 | 0 | 0.85 |
|  |  |  | 17 | 1 | 0 | Mixed cirrhosis-normal serum iron |  | 32 | 2 | 0 | 0.86 |
|  | **Starcevic** | 2006 | 137 | 10 | 0 | alcoholic cirrhosis | clinical / laboratory examination; ultrasonographic imaging | 62 | 4 | 0 | 0.80 |
|  |  |  | 137 | 10 | 0 |  |  | 327 | 22 | 1 | 0.34 |
|  | **Willis** | 2000 | 157 | 28 | 5 | mixed Cirrhosis with HCC, HH or diabetes | archive biopsy specimens | 27 | 4 | 3 | **<0.05** |
|  | **Yonal** | 2007 | 16 | 0 | 0 | cirrhosis child-pugh stage A | clinical / laboratory examination  child-pugh score | 136 | 2 | 0 | 0.93 |
|  |  |  | 30 | 0 | 0 | cirrhosis child-pugh stage B |  | 136 | 2 | 0 | 0.93 |
|  |  |  | 51 | 0 | 0 | cirrhosis child-pugh stage C |  | 136 | 2 | 0 | 0.93 |
| HCC | **Beckman** | 2000 | 12 | 4 | 1 | HCC with cirrhosis | liver biopsies and autopsies for HCC | 259 | 34 | 1 | 0.92 |
|  |  |  | 43 | 10 | 1 | HCC |  | 259 | 34 | 1 | 0.92 |
|  | **Boige** | 2003 | 126 | 7 | 0 | HCC with all cirrhosis | clinical /laboratory examination; ultrasonographic imaging and liver biopsy | 93 | 6 | 1 | 0.03 |
|  |  |  | 71 | 5 | 0 | HCC with alcoholic cirrhosis |  | 55 | 1 | 1 | **<0.05** |
|  |  |  | 38 | 1 | 0 | HCC with viral cirrhosis |  | 28 | 2 | 0 | 0.85 |
|  |  |  | 17 | 1 | 0 | HCC with other cirrhosis |  | 10 | 3 | 0 | 0.64 |
|  | **Campo** | 2001 | 23 | 0 | 0 | HCC | clinical/laboratory examination; ultrasonographic imaging and liver biopsy | 99 | 1 | 0 | 0.96 |
|  | **Cauza** | 2003 | 139 | 18 | 5 | HCC | clinical/laboratory examination; ultrasonographic imaging and liver biopsy | 440 | 47 | 0 | 0.26 |
|  |  |  | 139 | 18 | 5 | HCC |  | 163 | 16 | 5 | **<0.05** |
|  | **Ezzikouri** | 2008 | 95 | 1 | 0 | HCC | clinical/laboratory examination; and liver biopsy | 219 | 3 | 0 | 0.92 |
|  | **Gharib** | 2011 | 99 | 1 | 0 | HCC | clinical/laboratory examination | 98 | 2 | 0 | 0.92 |
|  | **Hellerbrand** | 2003 | 120 | 17 | 0 | HCC | clinical /laboratory examination; and liver biopsy | 120 | 6 | 0 | 0.78 |
|  |  |  | 120 | 17 | 0 | HCC |  | 103 | 4 | 0 | 0.84 |
|  | **Lauret** | 2002 | 34 | 9 | 0 | HCC with alcoholic cirrhosis | clinical/ laboratory examination; ultrasonographic imaging and liver biopsy | 130 | 6 | 0 | 0.79 |
|  |  |  | 31 | 3 | 0 | HCC with virus-related cirrhosis |  | 59 | 5 | 0 | 0.75 |
|  |  |  | 65 | 12 | 0 | HCC with all cirrhosis |  | 148 | 11 | 0 | 0.65 |
|  | **Nahon** | 2008 | 33 | 7 | 0 | HCC with alcoholic cirrhosis | clinical/ laboratory examination; ultrasonographic imaging and liver biopsy | 116 | 6 | 0 | 0.78 |
|  |  |  | 58 | 5 | 0 | HCC with HCV related cirrhosis |  | 64 | 12 | 0 | 0.45 |
|  | **Neghina** | 2009 | 5 | 0 | 0 | HCC with cirrhosis and HCV infection | clinical / laboratory examination | 10 | 2 | 4 | **<0.05** |
|  | **Racchi** | 1999 | 12 | 0 | 0 | HCC | liver biopsy | 119 | 11 | 0 | 0.61 |
|  | **Ropero** | 2007 | 183 | 12 | 1 | HCC | clinical/ laboratory examination; ultrasonographic/ct imaging and liver biopsy | 158 | 23 | 0 | 0.36 |
|  | **Shi** | 2005 | 47 | 3 | 6 | HBV-related HCC | clinical/ laboratory examination; ultrasonographic imaging and liver biopsy | 59 | 1 | 0 | 0.95 |
|  | **Willis** | 2005 | 119 | 17 | 8 | HCC | liver biopsy | 1331 | 168 | 9 | 0.15 |
|  | **Yonal** | 2007 | 19 | 0 | 0 | HCC | clinical / laboratory examination | 136 | 2 | 0 | 0.93 |

HWE: Hardy-Weinberg-Equilibrium; HBV: hepatitis B virus; HCV: hepatitis C virus; HH, hereditary haemochromatosis; NAFLD: non-alcoholic fatty liver disease; HCC, hepatocellular carcinoma; NASH: non-alcoholic steatohepatitis.

^&^Brunt EM, Janney CG, Di Bisceglie AM, Neuschwander-Tetri BA, Bacon BR. Nonalcoholic steatohepatitis: a proposal for grading and staging the histological lesions. Am J Gastroenterol. 1999;94(9):2467-2474. Epub 1999/09/14. doi: 10.1111/j.1572-0241.1999.01377.x. PubMed PMID: 10484010.
