# Supplementary material for: Association between the HFE C282Y, H63D Polymorphisms and the Risks of Non-Alcoholic Fatty Liver Disease, Liver Cirrhosis and Hepatocellular Carcinoma: An Updated Systematic Review and Meta-Analysis of 5,758 Cases and 14,741 Controls
Source: PLoS One. 2016 Sep 22;11(9):e0163423. doi: 10.1371/journal.pone.0163423 (PMC5033482; doi:10.1371/journal.pone.0163423)
Supplement: S6 Table — (DOCX) [file pone.0163423.s009.docx]

S6 Table Genotype distribution of HFE H63D polymorphism.

|  |  |  | Case | | |  |  | Control | | |  |
| --- | --- | --- | --- | --- | --- | --- | --- | --- | --- | --- | --- |
| Disease | **First author** | **Year** | **HH** | **HD** | **DD** | **Disease** | **Diagnostic method or definition** | **HH** | **HD** | **DD** | ***P*_HWE_** |
| NAFLD | **Bonkovsky** | 1999 | 18 | 16 | 2 | NASH | clinical/laboratory examination and histopathological analysis | 246 | 92 | 10 | 0.69 |
|  | **Chitturi** | 2002 | 34 | 7 | 1 | NASH | based on the criteria of Brunt et al^&^ | 92 | 32 | 1 | 0.32 |
|  | **Dhillon** | 2007 | 49 | 9 | 1 | NASH | clinical/laboratory examination; and liver biopsy | 88 | 11 | 1 | 0.34 |
|  | **George** | 1998 | 35 | 14 | 2 | NASH | clinical/laboratory examination; liver biopsy; predominantly macrovesicular steatosis with lobular inflammation. | 68 | 20 | 2 | 0.72 |
|  | **Lee** | 2010 | 107 | 17 | 1 | NAFLD | clinical/ laboratory examination; ultrasonographic imaging and liver biopsy | 205 | 16 | 0 | 0.58 |
|  | **Lee** | 2009 | 41 | 2 | 0 | NAFLD | self-reported questionnaire, clinical/ laboratory examination; ultrasonographic imaging | 402 | 39 | 0 | 0.33 |
|  | **Lin** | 2005 | 32 | 1 | 0 | NAFLD | clinical/ laboratory examination; ultrasonographic imaging | 121 | 4 | 0 | 0.86 |
|  | **Neri** | 2008 | 189 | 83 | 0 | NASH | clinical/laboratory examination; ultrasonographic imaging; liver biopsy, | 313 | 117 | 0 | **<0.05** |
|  | **Sikorska** | 2013 | 43 | 19 | 5 | NAFLD | clinical/laboratory examination; liver ultrasonographic imaging, liver biopsy; increased level of blood fatty acids without other liver damaging factors. | 131 | 50 | 10 | 0.08 |
|  | **Simsek** | 2006 | 17 | 12 | 1 | NASH | not reported | 2013 | 458 | 206 | **<0.05** |
|  | **Valenti** | 2003 | 95 | 36 | 3 | NAFLD | clinical/laboratory examination; ultrasonographic imaging and liver biopsy | 216 | 67 | 8 | 0.32 |
|  | **Valenti** | 2006 | 216 | 112 | 25 | NAFLD | clinical/laboratory examination; liver biopsy; other causes of liver disease were excluded. | 67 | 20 | 2 | 0.73 |
|  | **Valenti** | 2010 | 401 | 167 | 19 | NAFLD | clinical/laboratory examination; liver biopsy; | 122 | 55 | 7 | 0.80 |
|  | **Valenti** | 2012 | 142 | 65 | 9 | NAFLD | clinical /laboratory examination; liver biopsy; | 184 | 79 | 8 | 0.89 |
|  | **Yoneda** | 2010 | 57 | 1 | 0 | NASH | clinical/laboratory examination and liver biopsy | 20 | 0 | 0 | - |
|  | **Zamin** | 2006 | 23 | 5 | 1 | NASH | clinical/laboratory examination; liver biopsy | 15 | 5 | 0 | 0.52 |
|  |  |  | 23 | 5 | 1 | NASH |  | 17 | 3 | 0 | 0.72 |
| liver cirrhosis | **Beckman** | 2000 | 8 | 9 | 0 | cirrhosis with HCC | liver biopsies and autopsies for HCC; and verified cirrhosis was defined as the disseminated occurrence of pseudolobuli surrounded by connective tissue. | 229 | 59 | 6 | 0.35 |
|  | **Dhillon** | 2007 | 119 | 17 | 0 | cryptogenic cirrhosis | clinical/laboratory examination; and liver biopsy | 88 | 11 | 1 | 0.34 |
|  |  |  | 14 | 5 | 0 | HBV or HCV-cirrhosis |  | 88 | 11 | 1 | 0.34 |
|  | **Gharib** | 2011 | 81 | 18 | 1 | HCV-related cirrhosis | clinical /laboratory examination  Child-Pugh score | 72 | 27 | 1 | 0.37 |
|  | **Gleeson** | 2006 | 131 | 50 | 2 | presumed alcoholic cirrhosis | clinical /laboratory examination; ultrasonographic imaging and liver biopsy | 90 | 39 | 1 | 0.14 |
|  |  |  | 45 | 22 | 2 | biopsy proven alcoholic cirrhosis |  | 90 | 39 | 1 | 0.14 |
|  | **Hellerbrand** | 2003 | 83 | 23 | 1 | Mixed cirrhosis without HCC | clinical /laboratory examination;  liver biopsy for complicated cirrhoisis without HCC, such as cryptogenic cirrhosis | 94 | 29 | 3 | 0.67 |
|  |  |  | 83 | 23 | 1 |  |  | 108 | 27 | 2 | 0.83 |
|  | **Jain** | 2011 | 438 | 56 | 2 | all cirrhosis | clinical/ laboratory examination; ultrasonographic imaging and liver biopsy for the diagnosis | 456 | 46 | 0 | 0.28 |
|  |  |  | 59 | 14 | 1 | HBV-related cirrhosis |  | 456 | 46 | 0 | 0.28 |
|  |  |  | 46 | 4 | 0 | HCV-related cirrhosis |  | 456 | 46 | 0 | 0.28 |
|  |  |  | 86 | 6 | 0 | alcoholic cirrhosis without hepatitis |  | 456 | 46 | 0 | 0.28 |
|  |  |  | 29 | 9 | 0 | alcoholic cirrhosis with hepatitis |  | 456 | 46 | 0 | 0.28 |
|  |  |  | 218 | 23 | 1 | cryptogenic cirrhosis |  | 456 | 46 | 0 | 0.28 |
|  | **Jowkar** | 2011 | 13 | 1 | 0 | cryptogenic cirrhosis with Iron overload | clinical/ laboratory examination; waiting for a liver transplant | 2 | 1 | 0 | 0.73 |
|  |  |  | 65 | 21 | 0 | cryptogenic cirrhosis with Normal iron |  | 34 | 13 | 0 | 0.27 |
|  | **Lauret** | 2002 | 119 | 43 | 17 | alcoholic cirrhosis | ultrasonographic imaging and liver biopsy | 99 | 46 | 14 | **0.02** |
|  |  |  | 68 | 28 | 2 | HBV/HCV-related cirrhosis |  | 99 | 46 | 14 | **0.02** |
|  | **Mah** | 2005 | 22 | 3 | 0 | HBV-related cirrhosis | clinical/ laboratory examination; ultrasonographic imaging and liver biopsy | 47 | 2 | 0 | 0.88 |
|  |  |  | 10 | 0 | 1 | HCV-related cirrhosis |  | 47 | 2 | 0 | 0.88 |
|  | **Neghina** | 2009 | 8 | 1 | 0 | Mixed cirrhosis with/without HCC or HCV infection | clinical / laboratory examination | 10 | 2 | 0 | 0.75 |
|  | **Ozturk** | 2010 | 16 | 1 | 1 | cryptogenic cirrhosis | questionnaire, clinical/ laboratory examination | 109 | 30 | 2 | 0.97 |
|  | **Panigrahi** | 2006 | 24 | 6 | 1 | cryptogenic cirrhosis | clinical/ laboratory examination; ultrasonographic/MRI imaging | 68 | 6 | 0 | 0.72 |
|  | **Pfeiffenberger** | 2012 | 41 | 6 | 1 | cirrhosis with Wilson disease | clinical /laboratory examination,  and liver biopsy | 83 | 11 | 1 | 0.37 |
|  | **Sikorska** | 2011 | 33 | 9 | 1 | Mixed cirrhosis-elevated serum iron | clinical / laboratory examination,  and liver biopsy | 6 | 1 | 1 | 0.10 |
|  |  |  | 12 | 6 | 0 | Mixed cirrhosis-normal serum iron |  | 23 | 11 | 0 | 0.26 |
|  | **Starcevic** | 2006 | 97 | 47 | 3 | alcoholic cirrhosis | clinical / laboratory examination; ultrasonographic imaging | 50 | 15 | 1 | 0.92 |
|  |  |  | 97 | 47 | 3 |  |  | 267 | 71 | 12 | **0.01** |
|  | **Yonal** | 2007 | 12 | 4 | 0 | Cirrhosis child-pugh stage A | clinical / laboratory examination | 103 | 33 | 2 | 0.72 |
|  |  |  | 20 | 10 | 0 | Cirrhosis child-pugh stage B | Child-Pugh score | 103 | 33 | 2 | 0.72 |
|  |  |  | 41 | 8 | 2 | Cirrhosis child-pugh stage C |  | 103 | 33 | 2 | 0.72 |
| HCC | **Beckman** | 2000 | 8 | 9 | 0 | HCC with cirrhosis | liver biopsies and autopsies for HCC | 229 | 59 | 6 | 0.35 |
|  |  |  | 37 | 17 | 0 | HCC |  | 229 | 59 | 6 | 0.35 |
|  | **Boige** | 2003 | 92 | 41 | 0 | HCC with all cirrhosis | clinical /laboratory examination; ultrasonographic imaging and liver biopsy | 59 | 40 | 1 | **0.04** |
|  |  |  | 52 | 24 | 0 | HCC with alcoholic cirrhosis |  | 33 | 24 | 0 | **0.04** |
|  |  |  | 26 | 13 | 0 | HCC with viral cirrhosis |  | 18 | 11 | 1 | 0.66 |
|  |  |  | 14 | 4 | 0 | HCC with other cirrhosis |  | 8 | 5 | 0 | 0.39 |
|  | **Campo** | 2001 | 16 | 6 | 1 | HCC | clinical/laboratory examination; ultrasonographic imaging and liver biopsy | 65 | 32 | 3 | 0.69 |
|  | **Cauza** | 2003 | 128 | 31 | 3 | HCC | clinical/laboratory examination; ultrasonographic imaging and liver biopsy | 385 | 96 | 6 | 1.00 |
|  |  |  | 128 | 31 | 3 | HCC |  | 144 | 37 | 3 | 0.73 |
|  | **Ezzikouri** | 2008 | 59 | 34 | 3 | HCC | clinical/laboratory examination; and liver biopsy | 160 | 60 | 2 | 0.16 |
|  | **Gharib** | 2011 | 52 | 43 | 5 | HCC | clinical/laboratory examination | 72 | 27 | 1 | 0.37 |
|  | **Hellerbrand** | 2003 | 108 | 27 | 2 | HCC | clinical /laboratory examination; and liver biopsy | 94 | 29 | 3 | 0.67 |
|  |  |  | 108 | 27 | 2 | HCC |  | 83 | 23 | 1 | 0.67 |
|  | **Lauret** | 2002 | 52 | 25 | 0 | HCC with all cirrhosis | clinical/ laboratory examination; ultrasonographic imaging and liver biopsy | 99 | 46 | 14 | **0.02** |
|  |  |  | 29 | 14 | 0 | HCC with alcoholic cirrhosis |  | 90 | 29 | 17 | **<0.05** |
|  |  |  | 23 | 11 | 0 | HCC with virus-related cirrhosis |  | 45 | 17 | 2 | 0.80 |
|  | **Mah** | 2005 | 44 | 1 | 0 | HBV-related HCC | clinical/ laboratory examination; ultrasonographic imaging and liver biopsy | 47 | 2 | 0 | 0.88 |
|  |  |  | 25 | 1 | 0 | HCV-related HCC |  | 47 | 2 | 0 | 0.88 |
|  | **Motawi** | 2013 | 29 | 10 | 0 | HCV-related HCC | diagnosed by high alpha-fetoprotein levels | 32 | 8 | 0 | 0.48 |
|  |  |  | 29 | 10 | 0 |  |  | 30 | 10 | 0 | 0.37 |
|  | **Neghina** | 2009 | 5 | 0 | 0 | HCC with cirrhosis and HCV infection | clinical / laboratory examination | 13 | 3 | 0 | 0.68 |
|  | **Racchi** | 1999 | 9 | 3 | 0 | HCC | liver biopsy | 85 | 42 | 3 | 0.40 |
|  | **Ropero** | 2007 | 102 | 85 | 9 | HCC | clinical/ laboratory examination; ultrasonographic/CT imaging and liver biopsy | 124 | 52 | 5 | 0.87 |
|  | **Shi** | 2005 | 50 | 4 | 2 | HBV-related HCC | clinical/ laboratory examination; ultrasonographic imaging and liver biopsy | 56 | 3 | 1 | **<0.01** |
|  | **Yonal** | 2007 | 11 | 6 | 2 | HCC | clinical / laboratory examination | 103 | 33 | 2 | 0.72 |

HWE: Hardy-Weinberg-Equilibrium; HBV: hepatitis B virus; HCV: hepatitis C virus; HH, hereditary haemochromatosis; NAFLD: non-alcoholic fatty liver disease; HCC, hepatocellular carcinoma; NASH: non-alcoholic steatohepatitis.

^&^Brunt EM, Janney CG, Di Bisceglie AM, Neuschwander-Tetri BA, Bacon BR. Nonalcoholic steatohepatitis: a proposal for grading and staging the histological lesions. Am J Gastroenterol. 1999;94(9):2467-2474. Epub 1999/09/14. doi: 10.1111/j.1572-0241.1999.01377.x. PubMed PMID: 10484010.
