# Supplementary material for: Association between the HFE C282Y, H63D Polymorphisms and the Risks of Non-Alcoholic Fatty Liver Disease, Liver Cirrhosis and Hepatocellular Carcinoma: An Updated Systematic Review and Meta-Analysis of 5,758 Cases and 14,741 Controls
Source: PLoS One. 2016 Sep 22;11(9):e0163423. doi: 10.1371/journal.pone.0163423 (PMC5033482; doi:10.1371/journal.pone.0163423)
Supplement: S7 Table — (DOCX) [file pone.0163423.s010.docx]

S7 Table Subgroup analyses for HFE C282Y.

|  |  | homozygote  YY vs CC | | | heterozygote  CY vs CC | | | dominant  CY+YY vs CC | | | recessive  YY vs CC+CY | | | | | allele  Y vs C | | |
| --- | --- | --- | --- | --- | --- | --- | --- | --- | --- | --- | --- | --- | --- | --- | --- | --- | --- | --- |
| Disease | **Subgroup** | **N** | **OR**  **(95% CI)** | ***P_association_*** | **N** | **OR**  **(95% CI)** | ***P_association_*** | **N** | **OR**  **(95% CI)** | ***P_association_*** | **N** | **OR**  **(95% CI)** | ***P_association_*** | | | **N** | **OR**  **(95% CI)** | ***P_association_*** |
| NAFLD | **Ethnicity** |  |  |  |  |  |  |  |  |  |  |  |  | | |  |  |  |
|  | Caucasian | 7 | 5.83  (1.77~19.18) | **0.004** | 9 | 2.43  (1.43~4.13) | **0.001** | 9 | 2.60  (1.51~4.45) | **0.001** | 7 | 5.33  (1.69~16.79) | **0.004** | | | 9 | 2.60  (1.53~4.42) | **<0.001** |
|  | Asian | 1 | 0.16  (0.01~262) | 0.199 | 1 | 0.07  (0.00~1.18) | 0.065 | 1 | 0.05  (0.00~0.81) | **0.035** | 1 | 0.20  (0.01~3.22) | 0.254 | | | 1 | 0.04  (0.00~0.69) | **0.026** |
|  | Mixed | - | - | - | 2 | 0.75  (0.33~1.71) | 0.487 | 2 | 0.75  (0.33~1.71) | 0.487 | - | - | - | | | 2 | 0.80  (0.40~1.63) | 0.543 |
|  | **Source of controls** | | |  |  |  |  |  |  |  |  |  |  | |  | |  |  |
|  | PB | 7 | 4.39  (0.88~21.91) | 0.071 | 9 | 2.32  (1.26~4.27) | **0.007** | 9 | 2.49  (1.33~4.65) | **0.004** | 7 | 4.19  (0.97~18.10) | 0.055 | | 9 | | 2.53  (1.38~4.67) | **0.003** |
|  | HB | 1 | 0.41  (0.02~8.09) | 0.559 | 3 | 0.99  (0.54~1.81) | 0.965 | 3 | 0.92  (0.50~1.68) | 0.784 | 1 | 0.40  (0.02~7.82) | 0.545 | | 3 | | 0.88  (0.51~1.50) | 0.634 |
|  | **Genotyping method** | | |  |  |  |  |  |  |  |  |  |  | |  | |  |  |
|  | PCR-RFLP | 7 | 3.85  (0.71~20.89) | 0.118 | 9 | 1.78 (0.95~3.33) | 0.071 | 9 | 1.89  (0.97~3.66) | 0.060 | 7 | 3.71  (0.79~17.48) | 0.098 | | 9 | | 1.93  (1.00~3.72) | **0.049** |
|  | other | 1 | 0.91  (0.04~22.83) | 0.956 | 3 | 2.11 (0.71~6.24) | 0.177 | 3 | 2.06  (0.71~5.95) | 0.183 | 1 | 0.79  (0.03~19.60) | 0.884 | | 3 | | 1.93  (0.72~5.17) | 0.193 |
|  | **HWE** |  |  |  |  |  |  |  |  |  |  |  |  | |  | |  |  |
|  | *P_HWE_* >0.05 | 5 | 5.99  (1.53~23.47) | **0.010** | 9 | 2.08  (1.13~3.84) | **0.019** | 9 | 2.20  (1.19~4.07) | **0.012** | 5 | 5.43  (1.39~21.25) | **0.015** | | 9 | | 2.21  (1.23~3.98) | **0.008** |
|  | *P_HWE_* <0.05 | 3 | 1.12  (0.02~78.74) | 0.959 | 3 | 1.12  (0.28~4.48) | 0.874 | 3 | 0.94  (0.17~5.29) | 0.941 | 3 | 1.14  (0.02~54.56) | 0.947 | | 3 | | 0.81  (0.11~5.76) | 0.836 |
|  | **Disease type** | | |  |  |  |  |  |  |  |  |  |  | |  | |  |  |
|  | NASH | 4 | 3.05  (0.13~70.35) | 0.485 | 7 | 1.23  (0.68~2.26) | 0.493 | 7 | 1.25  (0.63~2.48) | 0.520 | 4 | 2.95  (0.17~50.57) | 0.455 | | 7 | | 1.26  (0.63~2.54) | 0.511 |
| liver cirrhosis | **Ethnicity** |  |  |  |  |  |  |  |  |  |  |  |  | |  | |  |  |
|  | Caucasian | 9 | 0.88  (0.41~1.91) | 0.751 | 17 | 0.95  (0.74~1.22) | 0.682 | 17 | 0.94  (0.74~1.20) | 0.603 | 9 | 0.87  (0.40~1.88) | 0.728 | | 17 | | 0.93  (0.74~1.17) | 0.542 |
|  | African | 0 | - | - | 1 | 0.49  (0.04~5.55) | 0.568 | 1 | 0.49  (0.04~5.55) | 0.568 | 0 | - | - | | 1 | | 0.50  (0.04~5.53) | 0.570 |
|  | Asian | 0 | - | - | 4 | 1.19  (0.28~5.04) | 0.811 | 4 | 1.19  (0.28~5.04) | 0.811 | 0 | - | - | | 4 | | 1.20  (0.29~5.07) | 0.801 |
|  | **Source of controls** | | |  |  |  |  |  |  |  |  |  |  | |  | |  |  |
|  | PB | 3 | 1.12  (0.23~5.42) | 0.885 | 13 | 1.07  (0.77~1.50) | 0.680 | 13 | 1.07  (0.77~1.49) | 0.704 | 3 | 1.11  (0.23~5.35) | 0.898 | | 13 | | 1.06  (0.77~1.46) | 0.732 |
|  | HB | 4 | 0.83  (0.29~2.41) | 0.732 | 7 | 0.64  (0.37~1.08) | 0.094 | 7 | 0.65  (0.40~1.07) | 0.091 | 4 | 0.82  (0.28~2.36) | 0.713 | | 7 | | 0.69  (0.44~1.07) | 0.099 |
|  | PB+HB | 2 | 0.80  (0.17~3.74) | 0.777 | 2 | 1.03  (0.63~1.68) | 0.914 | 2 | 1.01  (0.63~1.62) | 0.979 | 2 | 0.79  (0.17~3.69) | 0.769 | | 2 | | 0.99  (0.64~1.53) | 0.952 |
|  | **Genotyping method** | | |  |  |  |  |  |  |  |  |  |  | |  | |  |  |
|  | PCR-RFLP | 5 | 0.63  (0.22~1.84) | 0.401 | 18 | 0.94  (0.71~1.25) | 0.678 | 18 | 0.92  (0.69~1.21) | 0.543 | 5 | 0.62  (0.22~1.81) | 0.385 | | 18 | | 0.90  (0.69~1.17) | 0.424 |
|  | other | 4 | 1.29  (0.41~4.00) | 0.661 | 4 | 0.97  (0.60~1.55) | 0.893 | 4 | 0.99  (0.63~1.55) | 0.974 | 4 | 1.27  (0.41~3.93) | 0.674 | | 4 | | 1.02  (0.68~1.53) | 0.929 |
|  | **HWE** |  |  |  |  |  |  |  |  |  |  |  |  | |  | |  |  |
|  | *P_HWE_* >0.05 | 6 | 1.44  (0.50~4. 13) | 0.495 | 18 | 0.92  (0.71~1.19) | 0.524 | 18 | 0.94  (0.72~1.21) | 0.615 | 6 | 0.43  (0.50~4.09) | 0.501 | | 18 | | 0.96  (0.75~1.23) | 0.744 |
|  | *P_HWE_* <0.05 | 3 | 0.49  (0.15~1. 55) | 0.221 | 4 | 1.19  (0.59~2.40) | 0.629 | 4 | 0.94  (0.51~1.75) | 0.857 | 3 | 0.48  (0.15~1.51) | 0.208 | | 4 | | 0.81  (0.47~1.38) | 0.436 |
|  | **Cirrhosis type** | | |  |  |  |  |  |  |  |  |  |  | |  | |  |  |
|  | alcoholic | 4 | 0.66  (0.19~2.29) | 0.511 | 8 | 1.01  (0.74~1.36) | 0.968 | 8 | 0.98  (0.73~1.32) | 0.877 | 4 | 0.66  (0.19~2.27) | 0.504 | | 8 | | 0.95  (0.72~1. 26) | 0.725 |
|  | viral | - | - | - | 2 | 1.05  (0.44~2.53) | 0.909 | 2 | 1.05  (0.44~2.53) | 0.909 | - | - | - | | 2 | | 1.05  (0.44~2. 49) | 0.910 |
|  | other | 5 | 1.08  (0.39~2.94) | 0.884 | 12 | 0.79  (0.49~1.28) | 0.346 | 12 | 0.83  (0.53~1.30) | 0.412 | 5 | 1.06  (0.39~2.88) | 0.909 | | 12 | | 0.87  (0.58~1.31) | 0.504 |
| HCC | **Ethnicity** |  |  |  |  |  |  |  |  |  |  |  |  |  | | |  |  |
|  | Caucasian | 7 | 2.17  (0.44~10.70) | 0.341 | 18 | 1.47  (0.96~2.25) | 0.077 | 18 | 1.49  (0.99~2.27) | 0.059 | 7 | 2.18  (0.46~10.27) | 0.324 | 18 | | | 1.49  (1.00~2.22) | 0.051 |
|  | African | 0 | - | - | 2 | 0.62  (0.12~3.28) | 0.578 | 2 | 0.62  (0.12~3.28) | 0.578 | 0 | - | - | 2 | | | 0.63  (0.12~3.27) | 0.579 |
|  | Asian | 1 | 16.28 (0.89~296.43) | 0.059 | 2 | 2.64  (0.42~16.6)2) | 0.300 | 2 | 5.38  (0.73~39.60) | 0.099 | 1 | 15.57  (0.86~283.22) | 0.064 | 2 | | | 6.56  (0.52~82.82) | 0.146 |
|  | Mixed | 2 | 5.01 (0.16~153.47) | 0.356 | 2 | 1.25  (0.80~1.96) | 0.322 | 2 | 1.43  (0.95~2.16) | 0.084 | 2 | 4.90 (0.16~151.63) | 0.365 | 2 | | | 1.56 (1.05~2.31) | **0.027** |
|  | **Source of controls** | | |  |  |  |  |  |  |  |  |  |  | |  | |  |  |
|  | PB | 6 | 10.62  (4.87~23.14) | **<0.001** | 12 | 1.36  (0.93~1.99) | 0.111 | 12 | 1.63  (1.10~2.42) | **0.015** | 6 | 10.32 (4.74~22.45) | **<0.001** | | 12 | | 1.82 (1.23~2.68) | **0.003** |
|  | HB | 4 | 0.70  (0.25~1.98) | 0.505 | 11 | 1.46  (0.79~2.70) | 0.228 | 11 | 1.33  (0.72~2.46) | 0.363 | 4 | 0.70 (0.25~1.97) | 0.500 | | 11 | | 1.23 (0.68~2.21) | 0.492 |
|  | PB+HB | 0 | - | - | 1 | 0.77  (0.08~7.48) | 0.821 | 1 | 0.77  (0.08~7.48) | 0.821 | 0 | - | - | | 1 | | 0.77 (0.08~7.45) | 0.821 |
|  | **HWE** |  |  |  |  |  |  |  |  |  |  |  |  | |  | |  |  |
|  | *P_HWE_* >0.05 | 6 | 10.62  (4.87~23.14) | **<0.001** | 20 | 1.46  (1.00~2.14) | 0.051 | 20 | 1.64  (1.12~2.38) | **0.010** | 6 | 10.32 (4.74~22.45) | **<0.001** | | 20 | | 1.75 (1.22~2.50) | **0.002** |
|  | *P_HWE_* <0.05 | 4 | 0.70  (0.25~1.98) | 0.505 | 4 | 1.22  (0.69-2.16) 9~2.16) | 0.487 | 4 | 1.64  (1.12~2.38) | 0.693 | 4 | 0.70 (0.25~1.97) | 0.500 | | 4 | | 0.96 (0.53~1.73) | 0.881 |
|  | **Genotyping method** | | |  |  |  |  |  |  |  |  |  |  | |  | |  |  |
|  | PCR-RFLP | 8 | 3.20  (0.91~11.28) | 0.070 | 19 | 1.52  (1.05~2.20) | **0.025** | 19 | 1.61  (1.11~2.33) | **0.012** | 8 | 3.07 (0.89~10.62) | 0.077 | | 19 | | 1.63 (1.13~2.34) | **0.009** |
|  | other | 2 | 1.82  (0.03~126.61) | 0.781 | 5 | 1.08  (0.47~2.48) | 0.864 | 5 | 1.10  (0.44~2.77) | 0.841 | 2 | 2.01 (0.04~108.33) | 0.732 | | 5 | | 1.11 (0.41~2.96) | 0.838 |
|  | **Disease** | |  |  |  |  |  |  |  |  |  |  |  | |  | |  |  |
|  | **Cirrhosis (+)** | 4 | 0.80  (0.07~8.74) | 0.851 | 11 | 1.52  (0.82~2.84) | 0.187 | 11 | 1.41  (0.74~2.68) | 0.298 | 4 | 0.80 (0.08~7.79) | 0.844 | | 11 | | 1.31 (0.69~2.48) | 0.406 |
|  | **Cirrhosis (-)** | 6 | 5.67  (1.84~17.47) | **0.002** | 13 | 1.29  (0.91~1.82) | 0.155 | 13 | 1.49  (1.04~2.15) | **0.030** | 6 | 5.55 (1.80~17.11) | **0.003** | | 13 | | 1.63 (1.13~2.36) | **0.009** |

N: Number of studies; HWE: Hardy-Weinberg-Equilibrium; NAFLD: non-alcoholic fatty liver disease; HCC, hepatocellular carcinoma; NASH: non-alcoholic steatohepatitis; PCR-RFLP: polymerase chain reaction–restriction fragment length polymorphism; PB: population-based; HB: Hospital-based.
