# Supplementary material for: Association between the HFE C282Y, H63D Polymorphisms and the Risks of Non-Alcoholic Fatty Liver Disease, Liver Cirrhosis and Hepatocellular Carcinoma: An Updated Systematic Review and Meta-Analysis of 5,758 Cases and 14,741 Controls
Source: PLoS One. 2016 Sep 22;11(9):e0163423. doi: 10.1371/journal.pone.0163423 (PMC5033482; doi:10.1371/journal.pone.0163423)
Supplement: S8 Table — (DOCX) [file pone.0163423.s011.docx]

S8 Table Subgroup analyses for HFE H63D.

|  |  | | homozygote  DD vs HH | | | | heterozygote  HD vs HH | | | | | | | dominant  HD+DD vs HH | | | | | | recessive  DD vs HH+HD | | | | allele  D vs H | | | |
| --- | --- | --- | --- | --- | --- | --- | --- | --- | --- | --- | --- | --- | --- | --- | --- | --- | --- | --- | --- | --- | --- | --- | --- | --- | --- | --- | --- |
| Disease | **Subgroup** | | **N** | **OR**  **(95% CI)** | | ***P_association_*** | **N** | | **OR**  **(95% CI)** | | ***P_association_*** | | | **N** | **OR**  **(95% CI)** | | ***P_association_*** | | | **N** | **OR**  **(95% CI)** | ***P_association_*** | | **N** | **OR**  **(95% CI)** | ***P_association_*** | |
| NAFLD | **Ethnicity** | |  |  | |  |  | |  | |  | | |  |  | |  | | |  |  |  | |  |  |  | |
|  | Caucasian | | 8 | 1.48  (0.95~2.31) | | 0.086 | 9 | | 1.17  (0.99~1.37) | | 0.062 | | | 9 | 1.19  (1.02~1.39) | | **0.030** | | | 8 | 1.40  (0.90~2.18) | 0.138 | | 9 | 1.18 (1.03~1.35) | **0.018** | |
|  | Asian | | 3 | 1.20  (0.34~4.25) | | 0.775 | 6 | | 1.73  (1.15~2.61) | | **0.009** | | | 6 | 1.66  (1.11~2.48) | | **0.014** | | | 3 | 0.91  (0.26~3.10) | 0.874 | | 6 | 1.51 (1.05~2.17) | **0.028** | |
|  | Mixed | | 2 | 2.10  (0.21~21.12) | | 0.528 | 2 | | 0.87  (0.31~2.44) | | 0.791 | | | 2 | 1.04  (0.38~2.84) | | 0.934 | | | 2 | 2.16  (0.22~21.49) | 0.512 | | 2 | 1.24 (0.49~3.10) | 0.652 | |
|  | **Source of controls** | | | | |  |  | |  | |  | | |  |  | |  | | |  |  |  | |  |  |  | |
|  | PB | 10 | | 1.44  (0.91~2.26) | | 0.117 | 12 | | 1.25  (1.07~1.46) | | **0.006** | | | 12 | 1.26  (1.08~1.47) | | **0.003** | | | 10 | 1.31  (0.84~2.04) | 0.234 | | 12 | 1.22 (1.07~1.40) | **0.003** | |
|  | HB | 3 | | 1.64  (0.60~4.47) | | 0.336 | 5 | | 0.96  (0.58~1.56) | | 0.857 | | | 5 | 1.04  (0.66~1.66) | | 0.862 | | | 3 | 1.59  (0.59~4.28) | 0.358 | | 5 | 1.12 (0.75~1.67) | 0.582 | |
|  | **Genotyping method** | | | | |  |  | |  | |  | | |  |  | |  | | |  |  |  | |  |  |  | |
|  | PCR-RFLP | 11 | | 1.45  (0.91~2.30) | | 0.117 | 14 | | 1.31  (1.09~1.59) | | **0.005** | | | 14 | 1.33  (1.11~1.59) | | **0.002** | | | 11 | 1.31  (0.83~2.06) | 0.242 | | 14 | 1.28 (1.09~1.50) | **0.002** | |
|  | other | 2 | | 1.55  (0.62~3.90) | | 0.352 | 3 | | 1.07  (0.84~1.37) | | 0.583 | | | 3 | 1.09  (0.86~1.39) | | 0.477 | | | 2 | 1.54  (0.61~3.85) | 0.358 | | 3 | 1.10 (0.89~1.36) | 0.397 | |
|  | **HWE** |  | |  | |  |  | |  | |  | | |  |  | |  | | |  |  |  | |  |  |  | |
|  | *P_HWE_* >0.05 | 12 | | 1.55  (1.01~2.38) | | **0.046** | 14 | | 1.18  (0.99~1.40) | | 0.059 | | | 14 | 1.22  (1.03~1.43) | | **0.020** | | | 12 | 1.47  (0.96~2.24) | 0.077 | | 14 | 1.21 (1.07~1.38) | **0.008** | |
|  | *P_HWE_* <0.05 | 1 | | 0.57  (0.08~4.34) | | 0.591 | 3 | | 1.35  (0.99~1.83) | | 0.057 | | | 3 | 1.31  (0.96~1.77) | | 0.084 | | | 1 | 0.41  (0.06~3.05) | 0.387 | | 3 | 1.21 (1.05~1.40) | 0.169 | |
|  | **Disease type** |  | |  | |  |  | |  | |  | | |  |  | |  | | |  |  |  | |  |  |  | |
|  | NASH | 7 | | 1.60  (0.70~3.66) | | 0.265 | 9 | | 1.32  (1.04~1.67) | | **0.023** | | | 9 | 1.33  (1.05~1.67) | | **0.017** | | | 7 | 1.31  (0.59~2.91) | 0.507 | | 9 | 1.28  (1.04~1.57) | **0.020** | |
| liver cirrhosis | **Ethnicity** |  | |  | |  | |  |  | |  | |  | |  | |  | |  | |  |  |  | |  |  | |
|  | Caucasian | 11 | | 0.77  (0.48~1.23) | | 0.268 | | 13 | 1.16  (0.92~1.48) | | 0.209 | | 13 | | 1.12  (0.98~1.40) | | 0.336 | | 11 | | 0.76  (0.48~1.21) | 0.254 | 13 | | 1.06  (0.88~1.28) | 0.559 | |
|  | Asian | 11 | | 2.99  (1.35~6.62) | | **0.007** | | 17 | 1.27  (0.96~1.67) | | 0.095 | | 17 | | 1.30  (0.99~1.70) | | 0.060 | | 11 | | 2.95  (1.34~6.52) | **0.007** | 17 | | 1.31  (1.02~1.69) | **0.034** | |
|  | African | 1 | | 0.89  (0.05~14.47) | | 0.934 | | 1 | 0.59  (0.30~1.16) | | 0.129 | | 1 | | 0.60  (0.31~1.17) | | 0.135 | | 1 | | 1.00  (0.06~16.21) | 1.000 | 1 | | 0.66  (0.36~1.20) | 0.172 | |
|  | **Source of controls** | | | | |  | |  |  | |  | |  | |  | |  | |  | |  |  |  | |  |  | |
|  | PB | 18 | | 1.05  (0.69~1.60) | | 0.820 | | 24 | 1.22  (0.97~1.53) | | 0.083 | | 24 | | 1.21  (0.97~1.51) | | 0.088 | | 18 | | 1.04  (0.69~1.58) | 0.850 | 24 | | 0.69  (0.60~0.79) | 0.092 | |
|  | HB | 3 | | 0.69  (0.15~3.20) | | 0.631 | | 5 | 1.10  (0.68~1.76) | | 0.705 | | 5 | | 1.06  (0.67~1.68) | | 0.795 | | 3 | | 0.67  (0.14~3.11) | 0.606 | 5 | | 0.62  (0.40~0.95) | 0.825 | |
|  | PB+HB | 2 | | 2.31  (0.42~12.83) | | 0.337 | | 2 | 0.97  (0.65~1.43) | | 0.871 | | 2 | | 1.00  (0.68~1.47) | | 0.996 | | 2 | | 2.32  (0.42~12.80) | 0.335 | 2 | | 0.63  (0.44~0.90) | 0.936 | |
|  | **Genotyping method** | | | | |  | |  |  | |  | |  | |  | |  | |  | |  |  |  | |  |  | |
|  | PCR-RFLP | 20 | | | 1.00  (0.67~1.52) | 0.984 | | 27 | 1.21  (0.99~1.49) | | 0.066 | | 27 | | 1.19  (0.98~1.46) | | 0.083 | | 20 | | 0.99  (0.66~1.49) | 0.980 | 27 | | 1.16  (0.97~1.40) | 0.107 | |
|  | other | 3 | | | 2.24  (0.52~9.63) | 0.279 | | 4 | 0.97  (0.68~1.40) | | 0.889 | | 4 | | 1.01  (0.71~1.45) | | 0.944 | | 3 | | 2.23  (0.66~1.49) | 0.279 | 4 | | 1.05  (0.77~1.45) | 0.749 | |
|  | **HWE** |  | |  | |  | |  |  | |  | |  | |  | |  | |  | |  |  |  | |  | |  |
|  | *P_HWE_* >0.05 | 20 | | 1.75  (0.99~3.08) | | 0.053 | | 28 | 1.19  (0.98~1.45) | | 0.076 | | 28 | | 1.20 (0.99~1.45) | | 0.059 | | 20 | | 1.70  (0.97~2.99) | 0.064 | 28 | | 1.20  (1.01~1.42) | | **0.043** |
|  | *P_HWE_* <0.05 | 3 | | 0.67 (0.38~1.18) | | 0.162 | | 3 | 1.10  (0.63~1.91) | | 0.746 | | 3 | | 1.02 (0.61~1.70) | | 0.947 | | 3 | | 0.68  (0.39~1.19) | 0.175 | 3 | | 0.95  (0.62~1.45) | | 0.810 |
|  | **Cirrhosis type** | | | | |  | |  |  | | |  |  | |  | | |  |  | |  |  |  | |  | |  |
|  | cryptogenic | 4 | | 2.26  (0.60~8.54) | | 0.228 | | 6 | 1.03  (0.65~1.61) | | | 0.915 | 6 | | 1.05  (0.67~1.64) | | | 0.847 | 4 | | 2.28  (0.60~8.67) | 0.228 | 6 | | 1.08  (0.71~1.65) | | 0.718 |
|  | viral | 5 | | 0.74  (0.30~1.78) | | 0.498 | | 7 | 1.25  (0.73~2.13) | | | 0.412 | 7 | | 1.27  (0.71~2.27) | | | 0.419 | 5 | | 0.74  (0.31~1.79) | 0.506 | 7 | | 1.32  (0.72~2.40) | | 0.373 |
|  | alcoholic | 5 | | 1.06  (0.60~1.88) | | 0.847 | | 7 | 1.24  (0.86~1.78) | | | 0.253 | 7 | | 1.24  (0.89~1.73) | | | 0.213 | 5 | | 1.05  (0.60~1.85) | 0.865 | 7 | | 1.20  (0.92~1.56) | | 0.185 |
|  | other | 9 | | 1.16  (0.50~2.67) | | 0.729 | | 11 | 1.19  (0.92~1.53) | | | 0.192 | 11 | | 1.17  (0.93~1.48) | | | 0.188 | 9 | | 1.12  (0.49~2.57) | 0.784 | 11 | | 1.14  (0.92~1.41) | | 0.225 |
| HCC | **Ethnicity** | | |  | |  | |  |  |  | | |  | |  |  | | |  | |  |  |  | |  | |  |
|  | Caucasian | 12 | | 0.57  (0.32~1.01) | | 0.056 | | 15 | 1.08  (0.82~1.44) | 0.582 | | | 15 | | 1.00  (0.75~1.32) | 0.983 | | | 12 | | 0.52 (0.29~0.92) | **0.024** | 15 | | 0.93 (0.73~1.18) | | 0.533 |
|  | African | 2 | | 5.30  (1.32~21.22) | | **0.018** | | 4 | 1.64  (1.16~2.31) | **0.005** | | | 4 | | 1.72  (1.23~2.42) | **0.002** | | | 2 | | 4.29 (1.07~17.19) | **0.040** | 4 | | 1.64 (1.22~2.21) | | **0.001** |
|  | Asian | 2 | | 4.30  (0.81~22.72) | | 0.086 | | 4 | 1.37  (0.63~3.01) | 0.427 | | | 4 | | 1.64  (0.80~3.38) | 0.176 | | | 2 | | 4.03 (0.78~20.83) | 0.096 | 4 | | 1.84 (1.00~3.40) | | 0.051 |
|  | Mixed | 2 | | 1.32  (0.45~3.83) | | 0.609 | | 2 | 0.96  (0.68~1.35) | 0.813 | | | 2 | | 0.98  (0.70~1.37) | 0.920 | | | 2 | | 1.33 (0.46~3.86) | 0.598 | 2 | | 1.01 (0.75~1.36) | | 0.956 |
|  | **Source of controls** | | | | |  | |  |  |  | | |  | |  |  | | |  | |  |  |  | |  | |  |
|  | PB | 11 | | 1.23  (0.73~2.05) | | 0.434 | | 14 | 1.37  (1.05~1.80) | **0.021** | | | 14 | | 1.35  (1.01~1.80) | 0.041 | | | 11 | | 1.10 (0.66~1.85) | 0.707 | 14 | | 1.27 (0.97~1.65) | | 0.081 |
|  | HB | 6 | | 0.42  (0.17~1.03) | | 0.058 | | 10 | 0.87  (0.68~1.11) | 0.254 | | | 10 | | 0.82  (0.65~1.05) | 0.110 | | | 6 | | 0.40 (0.16~0.99) | **0.047** | 10 | | 0.81 (0.65~1.00) | | **0.048** |
|  | PB+HB | 1 | | 4.08  (0.66~24.95) | | 0.130 | | 1 | 1.54  (0.92~2.57) | 0.103 | | | 1 | | 1.62  (0.98~2.68) | 0.062 | | | 1 | | 3.55 (0.58~21.59) | 0.169 | 1 | | 1.56 (1.01~2.42) | | **0.046** |
|  | **HWE** |  | |  | |  | |  |  |  | | |  | |  |  | | |  | |  |  |  | |  | |  |
|  | *P_HWE_* >0.05 | 14 | | 1.63  (1.00~2.68) | | 0.051 | | 20 | 1.26  (1.02~1.55) | **0.035** | | | 20 | | 1.26  (1.02~1.57) | **0.035** | | | 14 | | 1.48 (0.91~2.43) | 0.117 | 20 | | 1.23 (1.03~1.47) | | **0.026** |
|  | *P_HWE_* <0.05 | 4 | | 0.19  (0.06~0.62) | | **0.006** | | 5 | 0.89  (0.63~1.24) | 0.478 | | | 5 | | 0.76  (0.56~1.04) | 0.083 | | | 4 | | 0.18 (0.06~0.58) | **0.004** | 5 | | 0.70 (0.54~0.92) | | **0.009** |
|  | **Genotyping method** | | | | |  | |  |  |  | | |  | |  |  | | |  | |  |  |  | |  | |  |
|  | PCR-RFLP | 17 | | 0.98  (0.64~1.51) | | 0.937 | | 23 | 1.18  (0.97~1.44) | 0.100 | | | 23 | | 1.14  (0.93~1.40) | 0.194 | | | 17 | | 0.89 (0.58~1.37) | 0.602 | 23 | | 1.09  (0.90~1.32) | | 0.370 |
|  | other | 1 | | 1.29  (0.06~26.83) | | 0.871 | | 2 | 0.61  (0.17~2.11) | 0.434 | | | 2 | | 0.57  (0.17~1.99) | 0.381 | | | 1 | | 1.46 (0.07~29.85) | 0.807 | 2 | | 0.59  (0.19~1.88) | | 0.373 |
|  | **Disease** |  | |  | |  | |  |  |  | | |  | |  |  | | |  | |  |  |  | |  | |  |
|  | **cirrhosis (+)** | 6 | | 0.16  (0.05~1.55) | | **0.003** | | 9 | 1.02  (0.68~1.52) | 0.928 | | | 9 | | 0.88  (0.61~1.25) | 0.470 | | | 6 | | 0.15 (0.05~0.52) | **0.002** | 9 | | 0.79 (0.59~1.05) | | **0.108** |
|  | **cirrhosis (-)** | 12 | | 1.86  (1.12~3.10) | | **0.017** | | 16 | 1.26  (1.04~1.54) | **0.021** | | | 16 | | 1.28  (1.04~1.58) | **0.018** | | | 12 | | 1.70 (1.02~2.84) | **0.042** | 16 | | 1.27 (1.06~1.52) | | **0.010** |

N: Number of studies; HWE: Hardy-Weinberg Equilibrium; NAFLD: non-alcoholic fatty liver disease; HCC, hepatocellular carcinoma; NASH: non-alcoholic steatohepatitis; PCR-RFLP: polymerase chain reaction–restriction fragment length polymorphism; PB: population-based; HB: Hospital-based.
