# Supplementary material for: Association between the HFE C282Y, H63D Polymorphisms and the Risks of Non-Alcoholic Fatty Liver Disease, Liver Cirrhosis and Hepatocellular Carcinoma: An Updated Systematic Review and Meta-Analysis of 5,758 Cases and 14,741 Controls
Source: PLoS One. 2016 Sep 22;11(9):e0163423. doi: 10.1371/journal.pone.0163423 (PMC5033482; doi:10.1371/journal.pone.0163423)
Supplement: S9 Table — (DOCX) [file pone.0163423.s012.docx]

S9 Table Pooled analysis of the association between the HFE C282Y+H63D genotype frequencies and the risks of NAFLD, liver cirrhosis, and HCC.

|  |  |  | Test of association | | Heterogeneity | |  | Begg’s test | | Egger’s test | |
| --- | --- | --- | --- | --- | --- | --- | --- | --- | --- | --- | --- |
| Disease | **Subgroup** | **Number of studies** | **OR (95% CI)** | ***P_association_*** | **I^2^** | ***P*_heterogeneity_** | **Model** | **z** | ***P_Begg_*** | **t** | ***P_Egger_*** |
| NAFLD | overall | 6 | 1.45(0.63~3.32) | 0.378 | 41.1 | 0.132 | R | 1.13 | 0.260 | -1.52 | 0.204 |
|  | PB | 3 | 2.18(0.96~4.97) | 0.064 | 21.9 | 0.278 |  |  |  |  |  |
|  | HB | 3 | 0.62(0.12~3.20) | 0.565 | 48.4 | 0.144 |  |  |  |  |  |
|  | Caucasian | 4 | 2.13(1.11~4.08) | **0.023** | 0.0 | 0.465 |  |  |  |  |  |
|  | Mixed | 2 | 0.24(0.04~1.26) | 0.090 | 0.0 | 0.684 |  |  |  |  |  |
|  | NASH | 3 | 0.61(0.18~2.01) | 0.415 | 26.7 | 0.256 |  |  |  |  |  |
| liver cirrhosis | overall | 10 | 0.86(0.50~1.48) | 0.582 | 5.5 | 0.391 | F | 0.36 | 0.721 | 0.44 | 0.674 |
|  | PB | 5 | 1.01(0.43~2.35) | 0.982 | 47.5 | 0.106 |  |  |  |  |  |
|  | PB+HB | 2 | 0.68(0.30~1.55) | 0.363 | 0.0 | 0.496 |  |  |  |  |  |
|  | HB | 3 | 1.14(0.24~5.46) | 0.873 | 0.0 | 0.788 |  |  |  |  |  |
|  | Caucasian | 9 | 0.82(0.47~1.43) | 0.483 | 11.3 | 0.341 |  |  |  |  |  |
| HCC | overall | 14 | 1.70(1.03~2.80) | **0.039** | 0.0 | 0.514 | F | 0.33 | 0.743 | 0.18 | 0.861 |
|  | PB | 8 | 1.59(0.87~2.91) | 0.129 | 0.0 | 0.459 |  |  |  |  |  |
|  | PB+HB | 6 | 1.95(0.77~4.92) | 0.160 | 7.5 | 0.368 |  |  |  |  |  |

NAFLD: non-alcoholic fatty liver disease; HCC, hepatocellular carcinoma; NASH: non-alcoholic steatohepatitis; PB: population-based; HB: Hospital-based.
